# Super-moiré spin textures in twisted two-dimensional antiferromagnets

**DOI:** 10.1038/s41565-025-02103-y

**Published:** 2026-02-02

**Authors:** King Cho Wong, Ruoming Peng, Eric Anderson, Jackson Ross, Bowen Yang, Meixin Cheng, Sreehari Jayaram, Malik Lenger, Xuankai Zhou, Yan Tung Kong, Takashi Taniguchi, Kenji Watanabe, Michael A. McGuire, Rainer Stöhr, Adam W. Tsen, Elton J. G. Santos, Xiaodong Xu, Jörg Wrachtrup

**Affiliations:** 1 3rd Physikalisches Institut, University of Stuttgart, Stuttgart, Germany; 2 Department of Physics, University of Washington, Seattle, WA USA; 3 Institute for Condensed Matter Physics and Complex Systems, The University of Edinburgh, Edinburgh, UK; 4 Institute for Quantum Computing, University of Waterloo, Walterloo, Ontario Canada; 5 Department of Physics and Astronomy, University of Waterloo, Waterloo, Ontario Canada; 6 Department of Chemistry, University of Waterloo, Waterloo, Ontario Canada; 7 Research Center for Materials Nanoarchitectonics, National Institute for Materials Science, Tsukuba, Japan; 8 Research Center for Electronic and Optical Materials, National Institute for Materials Science, Tsukuba, Japan; 9 Materials Science and Technology Division, Oak Ridge National Laboratory, Oak Ridge, TN USA; 10 Higgs Centre for Theoretical Physics, University of Edinburgh, Edinburgh, UK; 11 Donostia International Physics Center, Donostia-San Sebastián, Spain; 12 Department of Materials Science and Engineering, University of Washington, Seattle, WA USA; 13 Max Planck Institute for Solid State Research, Stuttgart, Germany

**Keywords:** Magnetic properties and materials, Surfaces, interfaces and thin films, Scanning probe microscopy

## Abstract

Stacking two-dimensional layered materials offers a platform to engineer electronic and magnetic states. In general, the resulting states—such as moiré magnetism—have a periodicity at the length scale of the moiré unit cell. Here we study magnetic order in twisted double-bilayer chromium triiodide by means of scanning nitrogen-vacancy microscopy. We observe long-range magnetic textures extending beyond the single moiré unit cell, which we dub a super-moiré magnetic state. At small twist angles, the size of the spontaneous magnetic texture increases with twist angle, opposite to the underlying moiré wavelength. The spin-texture size reaches a maximum of about 300 nm in 1.1° twisted devices, an order of magnitude larger than the underlying moiré wavelength, and vanishes at twist angles above 2°. The obtained magnetic field maps suggest the formation of antiferromagnetic Néel-type skyrmions spanning multiple moiré cells. The twist-angle-dependent study, combined with large-scale atomistic Monte Carlo simulations, suggests that the magnetic competition between the Dzyaloshinskii–Moriya interaction, magnetic anisotropy and exchange interactions—which all depend on the relative rotation of the layers—produces the topological textures that emerge in the super-moiré spin order.

## Abstract (web summary)

Scanning nitrogen-vacancy microscopy unveils super-moiré spin textures emerging in twisted double-bilayer CrI_3_ and provides real-space evidence of antiferromagnetic Néel-type skyrmions spanning multiple moiré cells.

## Main

Controlling magnetic interactions has led to the discovery of exotic phases^1,2^, including topological textures such as skyrmions^2,3^ and frustrated states such as spin glasses^4,5^, which pave the way for next-generation spintronic technologies^6,7^. Such non-trivial states typically arise from competition among multiple magnetic interactions, giving rise to unusual magnetic phenomena. For example, the coexistence of ferromagnetic (FM) and antiferromagnetic (AFM) couplings can induce strong frustration^8^, giving rise to spin-glass behaviour that can even host skyrmions^9,10^. Beyond intrinsic interactions, magnetic interactions can also be tailored, such as alternating deposited materials to stabilize new magnetic states^3^. Recent advances in synthetic antiferromagnets^11^ have demonstrated that engineering interlayer and interfacial interactions facilitates intriguing non-collinear and topological phases^12^.

Two-dimensional (2D) materials have opened new possibilities to control electronic and magnetic interactions^13–15^. In particular, stacking layered 2D materials with a small relative twist angle leads to the formation of moiré superlattices, which can dramatically alter the electronic band structure and give rise to a wide range of emergent phenomena—including correlated insulating states, topological phases, superconductivity and magnetism^16,17^. In twisted 2D magnetic bilayers, both theoretical and experimental studies have shown a correlation between the stacking order in a single moiré cell and their magnetic responses^18–21^, leading to complex magnetic states^22–27^. Notably, in the previous report on near-zero degree twisted CrI_3_ (ref. ^19^), scanning quantum microscopy was utilized to directly visualize moiré magnetism, revealing stacking-dependent AFM and FM domains in twisted bilayer and double trilayer CrI_3_.

While previous theoretical and experimental studies have primarily focused on interactions confined within a single moiré unit cell, this framework may no longer suffice when additional interactions are present. In multilayer moiré heterostructures, for example, the interference of two distinct moiré patterns can produce a secondary periodicity—known as a super-moiré potential—with a characteristic length scale larger than either moiré lattice^28–30^. In twisted magnetic bilayers, magnetic textures are typically understood in terms of interlayer interactions confined within a single moiré unit cell. However, additional contributions—such as intralayer exchange and interface-driven magnetic interactions—can strongly compete with the interlayer coupling^13–15^. These competing effects have been previously considered only within the moiré unit cell, where the magnetic textures are assumed to strictly follow the underlying lattice modulation. However, as the twist angle increases, the moiré unit cell becomes progressively smaller, forcing all magnetic interactions into a more confined region. In such a regime, it becomes conceivable that the magnetic texture will decouple from the small moiré length scale as suggested in micromagnetics^31^, giving rise to magnetic supercells whose size deviates from that of the moiré lattice.

Here, we used nitrogen vacancy (NV) scanning microscopy and atomistic Monte Carlo simulation to explore the nanoscale magnetic texture of twisted double-bilayer (tDB) CrI_3_ at various twist angles (≤2°). Notably, we find that the local magnetization patterns of our twisted devices do not reflect the moiré stacking as suggested by earlier reports^26,27^. Instead, we identified super-moiré AFM orders with a large characteristic length scale of hundreds of nanometres, far exceeding the lattice moiré length scale of a few tens of nanometres. These super-moiré patterns are driven by complex magnetic competition, in contrast with previous studies, where the super-moiré potentials arise from the interference between two distinct moiré patterns in twisted multilayer heterostructures. More importantly, our observations suggest the presence of AFM skyrmions in tDB CrI_3_ after a field-cooling process.

## Extended FM domains

**Fig. 1:**
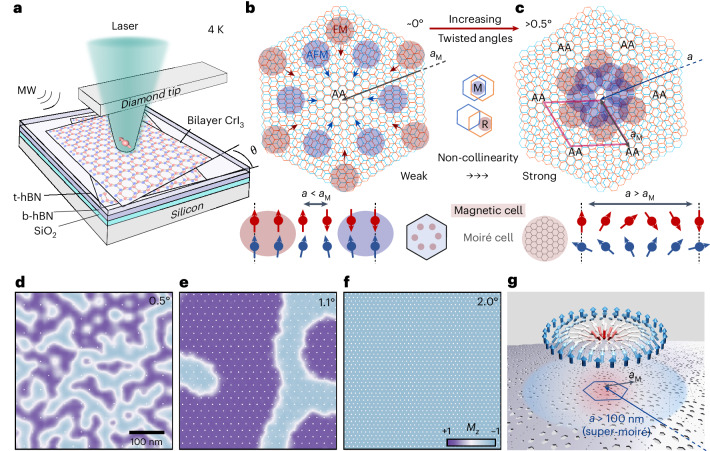
Evolution of competing magnetic orders in tDB CrI_3_. **a**, Schematic of the scanning quantum microscopy technique for the visualization of magnetic textures in tDB CrI_3_. An NV centre (red spin) is located at the apex of a diamond pillar. The NV is initialized by a green laser and controlled by a microwave (MW) signal. The sample device consists of two sheets of bilayer CrI_3_ with a twist angle *θ* between them. **b**, Schematic of moiré-modulated magnetic interactions at near-zero twist angle, showing the rhombohedral stacking region (R, shaded red) and monoclinic stacking region (M, shaded blue) are well separated. Thus, the magnetic texture closely follows the underlying moiré lattice, forming sharp magnetic domain walls. In this regime, *a* < *a*_M_, where *a* and *a*_M_ denote the length scale of magnetic texture and moiré wavelength, respectively. **c**, Schematic of competing magnetic orders at larger twist angles, where FM- and AFM-favoured regions shift towards AA sites. Magnetic competition drives strong non-collinear spin textures, with magnetic domains extending beyond a single moiré unit cell. In this regime, *a* > *a*_M_. **d**–**f**, Atomistic simulated normalized magnetization maps over a 450-nm region for twist angles of 0.5° (**d**), 1.1° (**e**) and 2° (**f**). Only magnetic competition of *J*_⊥_ and *J*_∥_ is considered. A cross-section of the magnetization from a single layer of the tDB CrI_3_ shows the merging of moiré cells into larger magnetic textures with increasing twist angle. White dots in the background mark the positions of underlying monoclinic sites for each twist angle. **g**, Schematic illustration of topological magnetic textures in relation to the moiré stacking. The arrows indicate the spin configuration in the 2D magnet, while the grey lattice beneath represents the moiré superlattice. The shaded holes artistically depict regions of distinct stacking. A representative moiré unit cell is outlined by a blue hexagon. The emergent super-moiré magnetic textures span multiple moiré unit cells, with characteristic length scales exceeding 100 nm.

Our magnetic systems consisted of two sheets of bilayer CrI_3_ with small twist angles, as illustrated in Fig. 1a. These twisted samples were fabricated using the tear-and-stack technique^32^ in an argon glovebox and were fully encapsulated with approximately 10-nm hexagonal boron nitride (hBN) flakes to prevent oxidation (Methods; Supplementary Sections 15–17). As a result of the twist, the local stacking of layers in tDB CrI_3_ alternates between monoclinic and rhombohedral configurations, leading to periodically modulated magnetic couplings. Specifically, the inner two layers (layers 2 and 3) exhibit alternating FM (rhombohedral stacking) and AFM (monoclinic stacking) coupling. Meanwhile, the interlayer interactions *J*_⊥_ of the outer bilayers (for example, layers 1 and 2; layers 3 and 4) are naturally AFM. The intralayer interactions *J*_∥_ of all four layers are expected to be FM. At near-zero twist angles, the FM and AFM order regions are well separated with each other within the moiré unit cell because the moiré wavelength is large, as shown in Fig. 1b. In this regime, *a* < *a*_M_, with *a* and *a*_M_ denoting the length scale of magnetic texture and moiré wavelength, respectively. The magnetic texture at this small angle limit is well described by a single-moiré-cell model, with the magnetization strictly following the underlying moiré superlattice stacking configuration and with sharp transitions between domains.

At a large twist angle, magnetic competition emerges as regions favouring interlayer FM and AFM couplings are brought closer together within a single moiré cell, as shown in Fig. 1c. In this regime, *a* > *a*_M_. Therefore, a rich landscape of magnetic phases can arise featuring emergent magnetization patterns, extended domain walls and non-collinear spin orders that span multiple moiré cells. We performed large-scale atomic simulations of tDB CrI_3_ with a record size of 450 nm, which consider only magnetic competition between *J*_⊥_ and *J*_∥_. At 0.5°, in one of the four layers, the simulations revealed large magnetic domains that span several moiré unit cells (Fig. 1d–f; see Supplementary Section 19 for simulation details). As the twist angle is increased to 1.1°, these domains expanded, and at 2°, the magnetization becomes nearly uniform across the entire 450-nm simulation area. Such angle-dependent behaviour is opposite to the predictions of the single moiré model, which anticipates shrinking magnetic textures with increasing twist angles. Instead, strong magnetic competition stabilizes domains with sizes larger than the moiré unit cell. Moreover, the presence of Dzyaloshinskii–Moriya interactions (DMI) and dipolar interactions induces canting in the spin configuration, resulting in extended magnetic textures. In particular, the uniform DMI induced by the hBN/CrI_3_ interface promotes the formation of Néel-type structures with length scales above 100 nm. The interplay between these competing magnetic interactions is crucial for stabilizing non-collinear and topological magnetic structures that exceed the moiré length scale, as illustrated in Fig. 1g.

**Fig. 2:**
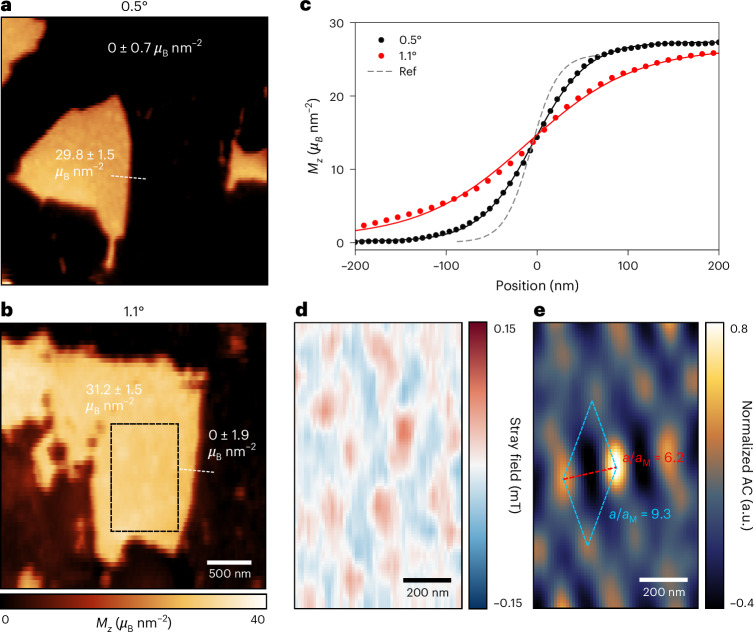
Emergent FM domain with correlated textures in tDB CrI_3_. **a**,**b**, 2D magnetization maps of tDB CrI_3_ at 0.5° and 1.1° twist angles, showing randomly distributed FM and AFM domains. The FM domains have a magnetization of around 30 *μ*_B_ nm^−^^2^, while other regions are all nearly 0 *μ*_B_ nm^−^^2^. **c**, Representative FM domain wall linecuts of 0.5° (red dots) and 1.1° (black dots) of tDB CrI_3_ samples, fitted with a hyperbolic tangent function (red and black lines). The fitting for 0.5° and 1.1° linecuts showed domain wall widths of 58.5 nm and 118.2 nm. Positions of the linecuts are denoted in **a** and **b** as dashed white lines. A domain wall linecut from a twisted bilayer CrI_3_ (dashed grey line) serves as a reference (Ref) for the minimum resolvable domain wall width of around 30 nm. See Supplementary Section 3 for more statistics and discussion. **d**, Stray field map of a selected FM area (dashed black rectangle) in **b**, after a smooth polynomial background subtraction, showing correlated field variation features within FM domains. **e**, 2D autocorrelation (AC) of the field map in **d**, revealing hexagonal textures with correlation length in the long edge around a = 340 nm, exceeding the moiré magnetic periodicity of 36.4 nm at a 1.1° twist angle, giving *a*/*a*_M_ ≈ 9.3. The short edge gives *a*/*a*_M_ ≈ 6.2.

To probe the underlying magnetic textures in tDB CrI_3_, we utilized NV scanning microscopy^33^ (Methods; Supplementary Section 1). Previous reports have shown that this technique offers a sensitivity of a few microteslas per square root hertz and a spatial resolution limited only by the spacing between the NV to sample distance. Compared with conventional optical approaches, NV scanning microscopy offers significantly improved spatial resolution and also allows direct reconstruction of local magnetization from detected stray field, as demonstrated in Fig. 2a,b. All measurements are performed at 4 K unless otherwise stated. From the magnetization maps of small-angle twisted (≤2°) samples (magnetization reconstruction is discussed in Supplementary Section 2), we distinguish two categories of magnetic response in the twisted regions: FM domains with a magnetization of approximately 30 *μ*_B_ nm^−^^2^ and AFM domains with a magnetization near 0 *μ*_B_ nm^−^^2^ (see also Supplementary Section 11). The uniform magnetization of ~30 *μ*_B_ nm^−^^2^ across the majority of FM regions corresponds to a net two-layer magnetization of CrI_3_ (refs. ^19,34^). Both FM and AFM domains are strongly pinned by local defects and remain stable over multiple thermal cycles.

Despite the moiré wavelengths at larger twist angles being smaller than our scanning spatial resolution of 30 nm, the detected FM domain magnetization significantly exceeds the expected maximum of 12 *μ*_B_ nm^−^^2^ based on a moiré unit cell model^26,27,35^. From our twist-angle-dependent study (discussed in Supplementary Sections 3 and 4), we find that the ratio of FM to AFM domain area follows a similar trend as previously reported reflective magnetic circular dichroism (RMCD) measurements^26^, exhibiting maximum FM response at 1.1°. We also notice a domain wall width between FM and AFM domains that is well above the spatial resolution of 30 nm, contrasting with the narrow domain wall width of below 10 nm expected in the single moiré model. The domain wall width also exhibits clear twist angle dependence, as illustrated in Fig. 2c. We identified the largest domain wall width of ~118 nm in 1.1° twisted samples, which are predicted to exhibit the largest FM responses and non-collinear features. The domain wall length far exceeds the moiré wavelength of the corresponding twist angle, which suggests that magnetic domains cannot be simply relaxed within a single moiré cell. This is also captured in our atomistic spin dynamics simulations, where the magnetic texture observed has a length scale larger than a moiré unit cell at 1.1°.

## Long-range AFM textures

In addition to the FM domain shown in Fig. 2a,b, we noticed small varying features inside these domains, which are shielded behind a strong stray field gradient generated from the FM-to-AFM domain boundaries. By applying a third-order polynomial background subtraction to eliminate the domain boundary field for the magnetic texture in Fig. 2b, we observed weak textures of about 100 μT, as shown in Fig. 2d (see more details in Supplementary Section 5). To confirm the underlying length scale, the autocorrelation of the 2D stray field map is computed as

1 $$\mathrm{AC}(\Delta x,\Delta y)=\mathop{\sum }\limits_{x,y}B(x,y)B(x+\Delta x,y+\Delta y),$$

where *B*(*x*, *y*) is the value of the 2D field map. This serves to reduce random noise caused by disorder and to enhance the underlying correlation in 2D maps^19,36^. The autocorrelation of the field map reveals hexagonal magnetic textures with a length scale reaching 340 nm, 9.3 times greater than the expected moiré magnetic wavelength of 36.4 nm for 1.1° twisted samples, giving *a*/*a*_M_ ≈ 9.3. Given the measured field strength, we attributed these observed textures to non-collinear AFM orders within one bilayer, coexisting with a uniform FM response in the other bilayer. In addition, similar weak magnetic textures were also observed in the FM region of 0.5° twisted samples with a length scale of 173 nm, giving *a*/*a*_M_ ≈ 2.4 (Supplementary Section 5). Interestingly, there is an inverse dependence of the magnetic length scale *a* on the underlying moiré wavelength *a*_M_.

**Fig. 3:**
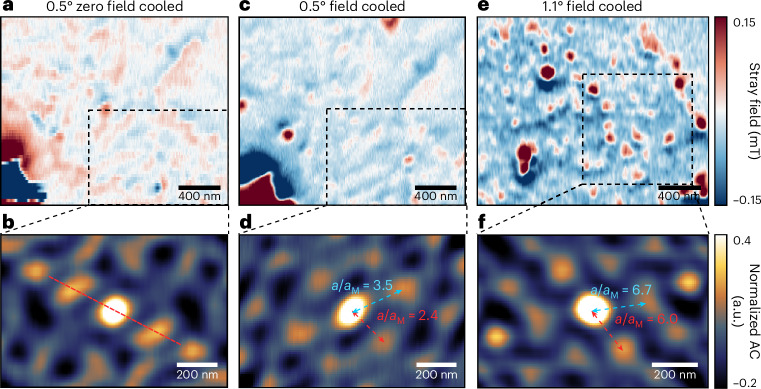
Long-range AFM textures with controlled cooling processes. **a**, A representative stray field map of 0.5° tDB CrI_3_ after zero-field cooldown, showing stripe-like patterns in an AFM region. **b**, Autocorrelation of a selected area (dashed black rectangle) in **a**, showing a one-dimensional correlation in the diagonal direction, highlighted by the dashed red line. **c**, Stray field map of the same sample area in **a** after 500-mT field cooldown, revealing dot-like patterns in the AFM region. **d**, Autocorrelation of the same area (dashed black rectangle) in **c**, showing a hexagonal texture with a spacing of *a*/*a*_M_ ≈ 3.5 in the long edge and *a*/*a*_M_ ≈ 2.4 in the short edge. **e**, Stray field map of 1.1° tDB CrI_3_ after 500-mT field cooling, showing dot-like patterns. **f**, Autocorrelation of a selected area (dashed black rectangle) in **e**, revealing a similar hexagonal pattern with a spacing of *a*/*a*_M_ ≈ 6.7 in the long edge and *a*/*a*_M_ ≈ 6.0 in the short edge.

A similar analysis can be applied to the AFM domains, as shown in Fig. 3. In this case, no background stray field subtraction is needed. In a 0.5° twisted sample, we observed a weak magnetic response at zero-field cooling with stripe-like patterns in the AFM domains (Fig. 3a), suggesting the presence of AFM textures within tDB regions. The autocorrelation of a selected area revealed a unilateral correlation in one direction (Fig. 3b). By contrast, no specific patterns are observed in the natural bilayer and four-layer regions (Supplementary Section 9). To enhance magnetic ordering, we applied field cooling at 0.5 T, a process developed in our previous twisted CrI_3_ experiments^19^. The observed AFM stripe-liked pattern transformed into dot-like pattern, as shown in Fig. 3c. Performing autocorrelation on the same selected area revealed a long-range hexagonal correlation (Fig. 3d), which suggests that the existence of DMI in the system contribute to the observed magnetic textures. We identified a wavelength of about 192 nm, giving *a*/*a*_M_ ≈ 2.4. We refer to this emergent texture of long-range magnetic order in twisted van der Waals layers as the super-moiré magnetic texture.

We examined AFM patterns at larger twist angles to explore the twist angle dependency. At 1.1°, we measured even larger super-moiré textures of about 218 nm, giving *a*/*a*_M_ ≈ 6.0 as shown in Fig. 3e,f. To corroborate these observations, more devices at these two angles were fabricated, consistently revealing a larger *a*/*a*_M_ at 1.1° than at 0.5° (Supplementary Section 6). A similar trend has also been observed for the super-moiré patterns in the FM regions of the sample (Supplementary Section 5). At angles greater than 1.1°, the expected moiré periodicity becomes smaller and non-collinear effects diminish, as also shown earlier^26^, leading to no discernible magnetic textures in 2° twisted samples (Supplementary Section 10).

## Super-moiré AFM skyrmions

**Fig. 4:**
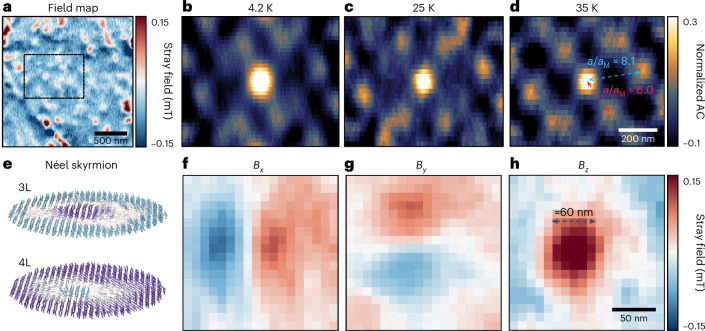
Emergence of AFM Néel-type skyrmions in tDB CrI_3_. **a**, A representative stray field map of a 1.1° tDB CrI_3_ sample after 500-mT field cooldown, taken at 4 K, shows dot-like features in the AFM region. An area (dashed black rectangle) was selected to perform autocorrelation at different temperatures. **b**–**d**, 2D autocorrelation of the selected area in **a** at 4 K (**b**), 25 K (**c**) and 35 K (**d**), demonstrating the robustness of the magnetic textures at elevated temperature. The contrast of higher-order peaks becomes more prominent due to the reduction of the critical field as the critical temperature is approached. The spacing of the hexagonal features yields a ratio of *a*/*a*_M_ ≈ 8.1 in the long edge and *a*/*a*_M_ ≈ 6.0 in the short edge. **e**, Atomistic simulation of tDB CrI_3_ (tDB CrI_3_), incorporating interfacial DMI from the hBN/CrI_3_ interface. Isolated AFM Néel-type skyrmions emerge in layers 3 and 4 (3L and 4L). Purple (blue) indicates out-of-plane spins pointing upwards (downwards), while white denotes in-plane spin orientation. **f**–**h**, *B*_*x*_ (**f**), *B*_*y*_ (**g**) and *B*_*z*_ (**h**) field reconstruction from fine scan *B*_NV_ of single dots in **a**. The field profile resembles the simulated Néel-type skyrmion shown in **e**. From the *B*_*z*_ profile, the feature size is about 60 nm.

With temperatures increasing from 4 K to 35 K, these super-moiré patterns in the twisted sample remain robust, preserving their super-moiré wavelength, as shown in Fig. 4a–d. A hexagonal correlation with *a/a*_M_ ≈ 8.1 is observed. The contrast of higher-order peaks became more prominent due to the reduction of the critical field at elevated temperatures. Furthermore, these super-moiré patterns were robust against magnetic fields, with the features becoming more prominent at higher applied fields (Supplementary Section 7). These signatures confirm the emergence of robust non-collinear magnetic textures with AFM orientations. Here, the moiré lattice and substrate effects can break the intrinsic symmetry of CrI_3_, introducing moiré and substrate DMI, and modulate the energy difference between various spin configurations. Under such conditions, the system can favour the formation of hexagonal patterns with skyrmion lattices. A more quantitative analysis of various magnetic energies, including magnetic anisotropy, exchange interaction, dipolar interaction and DMI, is presented in Supplementary Section 19, where isolated skyrmions and other main features such as extended AFM-type magnetic textures can emerge. However, due to the significantly increased complexity for larger simulation domains, the current simulation model^18,24^ cannot incorporate complex lattice relaxation^37,38^, such as magneto-elastic coupling^39–41^ and periodic lattice distortions^42^, which are typically observed in actual twisted systems. Such lattice relaxations can also contribute to the formation of super-moiré skyrmion lattices through the strong modulation of competing energies, requiring further theoretical studies of the lattice relaxation in magnetic systems.

To further confirm the topological nature of the skyrmion, we conducted zoomed-in scans on several of these small magnetic features. A representative scan is shown in Fig. 4f–h. The vector magnetic field map (*B*_*x*_, *B*_*y*_ and *B*_*z*_) was derived from the stray field map (*B*_NV_) detected by the NV probe (see also Supplementary Sections 2 and 12). It shows a skyrmion size of around 60 nm. Each single dot-like feature exhibits a magnetic field profile consistent with Néel skyrmions^43^, which are also observed in the simulation results shown in Fig. 4e. Atomic-scale simulations over a 450 × 450 nm area revealed that isolated Néel skyrmions can form in twisted samples. It is important to note that the skyrmions exhibit variations in size and shape, which we attribute to local strain and moiré disorder in the twisted samples. Such skyrmion lattices may contribute to a topological magnetic response, and the same region also exhibits a magnetic signal in RMCD measurements (Supplementary Fig. 30). This, in turn, could account for the observations in earlier RMCD and magneto-optical Kerr effect measurements, in addition to the net FM domains^44,45^. Our observations represent a direct visualization of skyrmion features in twisted 2D systems.

## Conclusions

We have utilized state-of-the-art scanning quantum microscopy to explore the nanoscale magnetic responses of tDB CrI_3_. Moving beyond the conventional framework of moiré magnetism, we identified super-moiré AFM order at small twist angles (for example, 0.5° and 1.1°), arising from the interplay between the twisted moiré potential and complex magnetic competition. More importantly, we uncovered the emergence of super-moiré magnetic textures with length scale inversely dependent on the underlying moiré wavelength. These textures also show the signatures of Néel-type skyrmions, which have not been reported in any twisted magnetic systems so far. These long-range magnetic textures can also appear in twisted double trilayers (Supplementary Section 10) and may even be present in more general twisted devices, reshaping earlier theoretical models^18,21^. These call for further investigation to unravel the emergence of super-moiré magnetic textures and other stacking-related behaviours, such as locally enhanced transition temperatures^34,46^ and rotational stacking faults that affect spin ordering^47^.

Our observations also enrich the understanding of 2D magnetism, in which strong magnetic anisotropy was believed to be required to stabilize long-range magnetic order, conditions generally considered unfavourable for the formation of topological magnetic textures such as skyrmion lattices. However, in twisted systems, the moiré effect introduces intense magnetic competition that not only stabilizes topological spin textures, but also preserves local single-ion anisotropy, thereby reconciling these contradictory requirements. In addition, our investigations can be generalized to a range of 2D magnetic families (CrBr_3_, CrCl_3_, CrSBr, Fe_5_GeTe_2_ and so on)^48–54^ that host very different interaction strengths and symmetries. Moiré engineering provides unconventional tuning knobs to manipulate multiple magnetic interactions with complex electronic competitions to drive the development of skyrmionic devices^55^. In this context, it may enable the discovery of other exotic magnetic phases such as half-charge merons^56^, topological magnons^57^ and quantum spin liquids^58^ driven by the moiré effect.

## Methods

### Experimental set-up

NV scanning probe measurements were performed in an attoLiquid1000 liquid-helium bath cryostat (Attocube Systems) equipped with a three-axis superconducting vector magnet (up to 0.5 T per axis). The system has a base temperature of 4.2 K, with a resistive heater beneath the sample holder, enabling temperature control up to 70 K. Scanning and positioning are achieved using integrated three-axis piezo scanners and positioners controlled by ASC500 and ANC350 units (Attocube). An Akiyama-based diamond probe (Qzabre) was used for NV scanning. The confocal microscope uses a 515-nm excitation laser passing through an acousto-optical modulator, single-mode fibre and 590-nm dichroic mirror and is focused onto the sample via a low-temperature objective (LT-APO/532-RAMAN/0.82, Attocube). NV photoluminescence is collected through the same optical path, directed into a multimode fibre and detected by an avalanche photodiode, while an integrated charge-coupled device camera enables real-time visualization of the tip and sample.

### Device fabrication

tDB CrI_3_ heterostructures were fabricated using a tear-and-stack technique to precisely control the twist angle. A thin top hBN layer (~10 nm) was first picked up using a polycarbonate (PC)/polydimethylsiloxane (PDMS) stamp. Exfoliated bilayer CrI_3_ flakes were then torn into two halves; one half was picked up and the other half was rotated by the desired angle (typically with <0.1° misalignment) before being restacked to form the twisted bilayer. Finally, a bottom hBN layer was also picked up to encapsulate the CrI_3_ stack. All stacking steps were performed inside an argon-filled glovebox to prevent sample degradation. The completed heterostructure was subsequently transferred adjacent to a predesigned planar microwave waveguide on a SiO_2_/Si substrate, which enables efficient microwave delivery for NV spin control during low-temperature scanning measurements.

## Online content

Any methods, additional references, Nature Portfolio reporting summaries, source data, extended data, supplementary information, acknowledgements, peer review information; details of author contributions and competing interests; and statements of data and code availability are available at 10.1038/s41565-025-02103-y.

## Supplementary information

Supplementary Information Supplementary Figs. 1–53, Tables 1 and 2 and Discussions.

## Data Availability

The data that support the findings of this study are available via Zenodo at 10.5281/zenodo.17545114 (ref. ^59^).
