# Supplementary material for: Super-moiré spin textures in twisted two-dimensional antiferromagnets
Source: Nat Nanotechnol. 2026 Feb 2;21(3):359–65. doi: 10.1038/s41565-025-02103-y (PMC13017526; doi:10.1038/s41565-025-02103-y)
Supplement: Supplementary file 1 — Supplementary Figs. 1–53, Tables 1 and 2 and Discussions. [file 41565_2025_2103_MOESM1_ESM.pdf]

nature nanotechnology

Supplementary information

https://doi.org/10.1038/s41565-025-02103-y

Super-moiré spin textures in twisted two-dimensional antiferromagnets

In the format provided by the authors and unedited

Contents

1 NV magnetometry based on the two-point method **3**

2 Vector field and magnetization reconstruction **5**

3 Angle Dependence of the Domain Wall Width **8**

4 Angle Dependence of the Net Magnetization **10**

5 Autocorrelation in FM domains **11**

5.1 Background subtraction of the FM domain in 0.5° tDB CrI_3 11

5.2 Autocorrelation of the FM domain in 0.5° tDB CrI_3 12

5.3 Background subtraction of the FM domain in 1.1° tDB CrI_3 13

5.4 Autocorrelation of the FM domain in 1.1° tDB CrI_3 14

6 Additional autocorrelation in the AFM domains **15**

7 Magnetic field dependence measurement of AFM domain **20**

8 The Stray field map at the edge of 0.5° tDB CrI_3 **22**

9 Pristine 4L and 2L CrI_3 devices **23**

10 Stray field maps of 1.2° tDT and 2.0° tDB CrI_3 **24**

11 Histogram of magnetization in tDB CrI_3 **25**

12 Additional magnetic field profile of the dot-like feature **28**

13 Short summary of the magnetic features observed in all tDB CrI_3 samples **33**

14 Determination of sample to NV distance **34**

15 Optical and AFM images of the tDB CrI_3 devices **35**

16 Optical images of a 1.1° tDB CrI_3 devices and the RMCD measurement **38**

17 AFM images of the hBN flakes used to encapsulate sample devices **39**

18 Stray field map and topography comparison 01 **40**

19 Simulation of tDB CrI_3 **48**

19.1 Method 48

19.2 Results 53

19.2.1 Moiré Spin Textures 53

19.2.2 Moiré Phases 56

19.2.3 Skyrmion Formation 62

1 NV magnetometry based on the two-point method

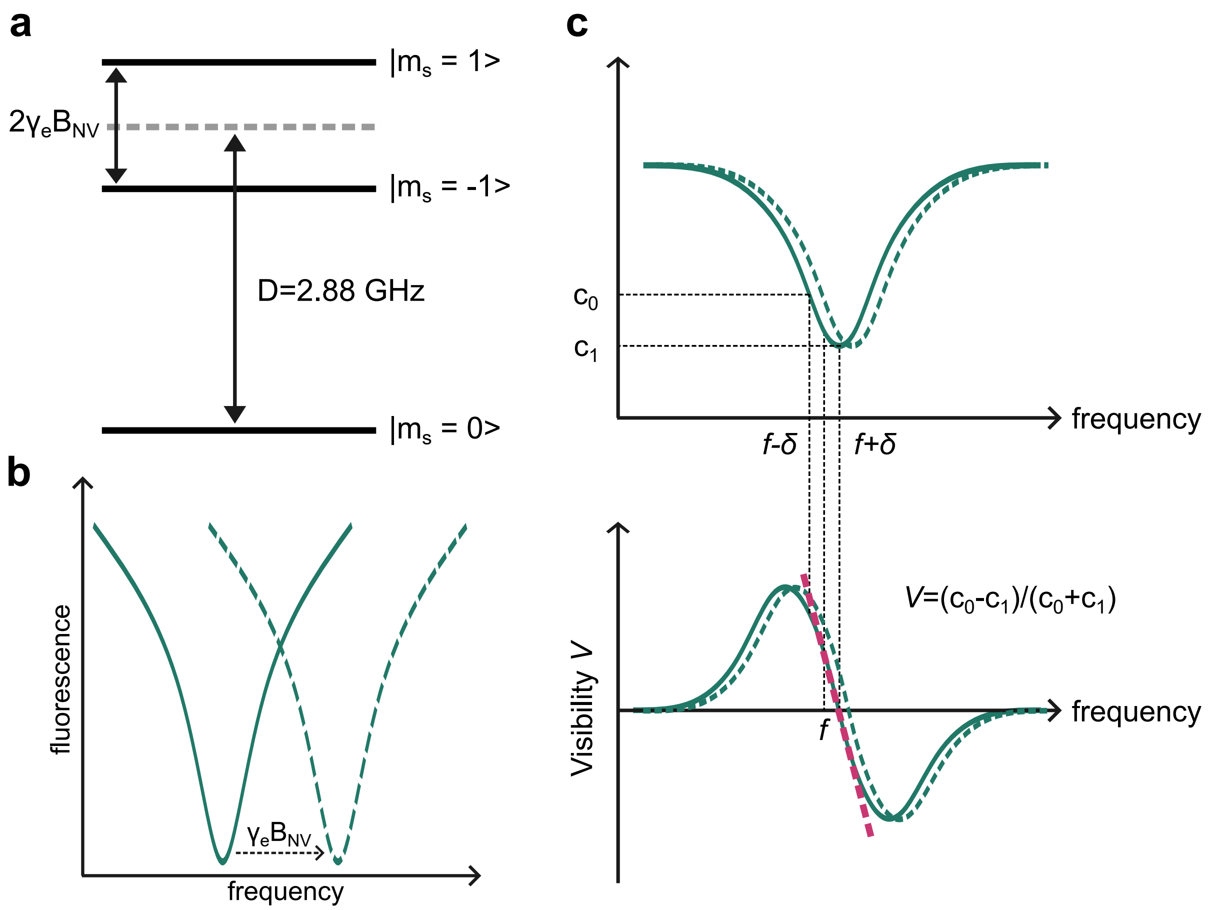

Figure S 1: **a**. Energy level of the electronic ground states of NV^- center. **b**. Example of an ODMR peak shift due to the Zeeman effect. **c**. The schematics of the two-point measurement method. The red dashed line denotes the linear regime of the visibility curve.

The negatively charged nitrogen-vacancy (NV) center in diamond has a spin-1 triplet electronic ground state (*1*), as shown in Figure S1(a). At zero magnetic field and 4 K, the $|m_s = 0\rangle$ state is separated from the degenerate $|m_s = \pm 1\rangle$ states by a zero-field splitting of $D \approx 2.88$ GHz. Applying a magnetic field along the NV axis lifts this degeneracy via the Zeeman effect, in the first order approximation, shifting the transition frequencies to $D \pm \gamma_e B_{NV}$, where $\gamma_e = 28$ GHz/T is the NV electron spin gyromagnetic ratio.

The NV centre shows spin-dependent fluorescence under green laser excitation. The $|m_s = \pm 1\rangle$ states undergo non-radiative decay through an intersystem crossing, leading to reduced photoluminescence compared to $|m_s = 0\rangle$. A green laser both initializes the spin state and enables optical readout. When microwave radiation drives transitions between $|m_s = 0\rangle$ and $|m_s = \pm 1\rangle$, a drop in fluorescence occurs at resonance, allowing B_{NV} to be measured via optically detected magnetic resonance (ODMR), as illustrated in Figure S1(b).

While full ODMR spectra can be measured at each pixel to construct a 2D stray field map, this is very time-consuming. Instead, we used a faster "two-point method", shown in Figure S1(c). At each pixel, fluorescence counts c_0 and c_1 are recorded at two frequencies, $f - \delta$ and $f + \delta$, where δ is the half-width-at-half-maximum (HWHM) of the ODMR peak. The visibility is calculated as $V = (c_0 - c_1)/(c_0 + c_1)$, which varies approximately linearly near the resonance frequency (S1(c)). The sign of V indicates the direction of the frequency shift, and its magnitude reflects the shift length. This method allows rapid field mapping with minimal loss of sensitivity. If the shift in resonance frequency of the ODMR signal at different pixel is small enough, roughly smaller then the FWHM of the ODMR peak, then this method could track the ODMR resonance frequency nicely.

2 Vector field and magnetization reconstruction

Consider a magnetic source located at or below the plane $z = 0$, such that the region $z > 0$ is free of sources. In the magnetostatic limit and in free space, the magnetic field satisfies (*2*):

$$\nabla \cdot \boldsymbol{B} = 0, \quad \nabla \times \boldsymbol{B} = 0. \tag{1}$$

In Cartesian coordinates, the curl-free condition yields:

$$\frac{\partial B_z}{\partial y} - \frac{\partial B_y}{\partial z} = 0, \tag{2}$$

$$\frac{\partial B_z}{\partial x} - \frac{\partial B_x}{\partial z} = 0, \tag{3}$$

$$\frac{\partial B_x}{\partial y} - \frac{\partial B_y}{\partial x} = 0. \tag{4}$$

Applying a 2D Fourier transform in the (x, y) plane transforms the magnetic field components into k-space:

$$\tilde{B}_i(k_x, k_y, z) = \int B_i(x, y, z)\, e^{-i(k_x x + k_y y)}\, dx\, dy, \tag{5}$$

$$B_i(x, y, z) = \frac{1}{(2\pi)^2} \int \tilde{B}_i(k_x, k_y, z)\, e^{i(k_x x + k_y y)}\, dk_x\, dk_y. \tag{6}$$

In Fourier space, Eqs. (2)–(4) become:

$$ik_y \tilde{B}_z - \frac{\partial \tilde{B}_y}{\partial z} = 0, \tag{7}$$

$$ik_x \tilde{B}_z - \frac{\partial \tilde{B}_x}{\partial z} = 0, \tag{8}$$

$$ik_y \tilde{B}_x - ik_x \tilde{B}_y = 0. \tag{9}$$

In a source-free region, the magnetic field decays exponentially with z, i.e., $\tilde{B}_i(z + \delta z) \approx \tilde{B}_i(z) e^{-k\delta z}$, where $k = \sqrt{k_x^2 + k_y^2}$. Substituting into Eq. (7) and evaluating the derivative gives:

$$\tilde{B}_y = -\frac{ik_y}{k} \tilde{B}_z. \tag{10}$$

A similar expression follows from Eq. (8):

$$\tilde{B}_x = -\frac{ik_x}{k}\tilde{B}_z. \tag{11}$$

These relations fully determine the magnetic field components in k-space. The projection onto the NV axis is then:

$$\tilde{B}_{NV} = \cos\phi\sin\theta\,\tilde{B}_x + \sin\phi\sin\theta\,\tilde{B}_y + \cos\theta\,\tilde{B}_z. \tag{12}$$

Substituting Eqs. (11) and (10) into Eq. (12) allows expressing $\tilde{B}_{NV}$ solely in terms of $\tilde{B}_z$, and thereby inverting to obtain $\tilde{B}_z$ or $\tilde{M}_z$.

Now consider a magnetized film described by a 2D magnetization distribution $\boldsymbol{M}(\boldsymbol{r'})$ lying in the $z = 0$ plane. The magnetic field at position $\boldsymbol{r}$ is given by:

$$\boldsymbol{B}(\boldsymbol{r}) = \frac{\mu_0}{4\pi}\int_A \left[\frac{3(\boldsymbol{M}\cdot\boldsymbol{r''})\boldsymbol{r''}}{|\boldsymbol{r''}|^5} - \frac{\boldsymbol{M}}{|\boldsymbol{r''}|^3}\right] d^2\boldsymbol{r'}, \tag{13}$$

where $\boldsymbol{r''} = \boldsymbol{r} - \boldsymbol{r'}$.

Assuming $\boldsymbol{M}(\boldsymbol{r'}) = M_j(\boldsymbol{r'})\hat{e}_j$, the field becomes:

$$B_i(\boldsymbol{r}) = \int_A M_j(\boldsymbol{r'})G_{ij}(\boldsymbol{r}-\boldsymbol{r'})\,d^2\boldsymbol{r'}, \tag{14}$$

where G_{ij} is the real-space Green's function. Taking the 2D Fourier transform yields:

$$\tilde{B}_i(k_x,k_y,z) = \tilde{M}_j(k_x,k_y)\,\tilde{G}_{ij}(k_x,k_y,h), \tag{15}$$

with $h = z - z'$ and

$$\tilde{G}_{ij}(k_x,k_y,h) = \frac{1}{2}\mu_0 k e^{-kh}\begin{bmatrix} -\frac{k_x^2}{k^2} & -\frac{ik_xk_y}{k^2} & -\frac{ik_x}{k} \\ -\frac{ik_xk_y}{k^2} & -\frac{k_y^2}{k^2} & -\frac{ik_y}{k} \\ -\frac{ik_x}{k} & -\frac{ik_y}{k} & 1 \end{bmatrix}. \tag{16}$$

If the magnetization is purely out-of-plane, i.e., $\boldsymbol{M} = M_z\hat{z}$, this simplifies to:

$$\begin{bmatrix} \tilde{B}_x \\ \tilde{B}_y \\ \tilde{B}_z \end{bmatrix} = \frac{1}{2}\mu_0 k e^{-kh}\begin{bmatrix} -\frac{ik_x}{k} \\ -\frac{ik_y}{k} \\ 1 \end{bmatrix}\tilde{M}_z. \tag{17}$$

Combining this with Eq. (12), we express $\tilde{M}_z$ in terms of $\tilde{B}_{NV}$:

$$\tilde{M}_z = \frac{\tilde{B}_{NV}}{\frac{1}{2}\mu_0 k e^{-kh}\left(-i\frac{k_x}{k}\cos\phi\sin\theta - i\frac{k_y}{k}\sin\phi\sin\theta + \cos\theta\right)}. \tag{18}$$

This expression enables reconstruction of $M_z(x, y)$ from the measured $B_{NV}(x, y)$ via inverse Fourier transform, provided the NV–sample distance h is known.

To determine h, we fit the NV-measured B_{NV} profile at a ferromagnetic domain sample edge using:

$$B_z(x) \approx -\frac{\mu_0 m_z x}{2\pi(x^2 + h^2)}, \tag{19}$$

$$B_x(x) \approx \frac{\mu_0 m_z h}{2\pi(x^2 + h^2)}, \tag{20}$$

$$B_{NV}(x) = B_x(x)\cos\phi\sin\theta + B_z(x)\cos\theta \tag{21}$$

where m_z is the magnetic moment density.

3 Angle Dependence of the Domain Wall Width

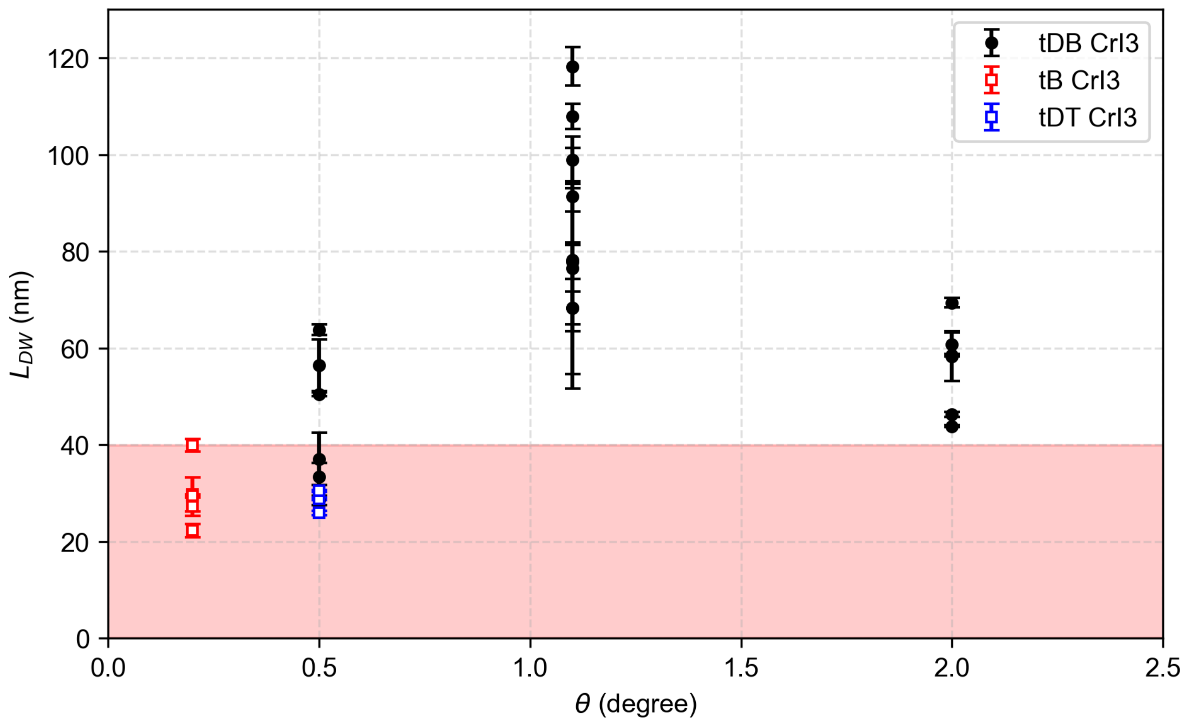

Figure S 2: Domain wall width statistics from twisted double bilayer (tDB) (solid black dot), twisted bilayer (tB) (hollow red square) and twisted double trilayer (tDT) (hollow blue square) CrI_3 at various twist angles. Each data point represents a particular FM-to-AFM domain wall from the respective samples. The value is extracted by fitting the domain wall linecut into the function $M_z(r) = A_0 \tanh(r/w)$, where w is defined as the wall width. The error bars represent the fitting error of w. It is observed that the domain wall from the $1.1°$ sample could go up to roughly 120 nm. The data point from tB and tDT domain wall definite the effective spatial resolution limit of the NV scanning probe, which is around 30 nm.

As discussed in the main text, twisted double bilayer (tDB) CrI_3 exhibits finite-width magnetic domain walls that can be spatially resolved using scanning quantum microscopy. The domain wall width is extracted by fitting the B-field linecut across a domain wall with a hyperbolic tangent function, $M_z(r) = A_0 \tanh(r/w)$, where w is the wall width. Error bar refers to the fitting error. Measurements across eight different samples reveal a clear dependence of

the domain wall width on the twist angle, reaching a maximum of approximately 120 nm at a twist angle of 1.1°. At this angle, the domain wall width exceeds the size of a single Moiré unit cell, allowing magnetic textures to extend across multiple Moiré cells as the system relaxes its magnetization. This broadened domain wall width at 1.1° may also be related to previously reported nonlinear magnetic phases, which show pronounced features near the same twist angle. For comparison, we performed a similar analysis on twisted bilayer (tB) CrI_3 and twisted double trilayer (tDT) CrI_3 devices with small twist angles (<0.5°). In these devices, the domain walls were expected to be less than 10 nm. However, our NV scanning probe captured wall width between 22-40 nm, with average of roughly 30 nm. This range essentially defines our spatial resolution. The red shaded shows region lower than 40 nm of width. We could therefore estimate that the spatial resolution of the NV scanning probe is around 30 nm.

4 Angle Dependence of the Net Magnetization

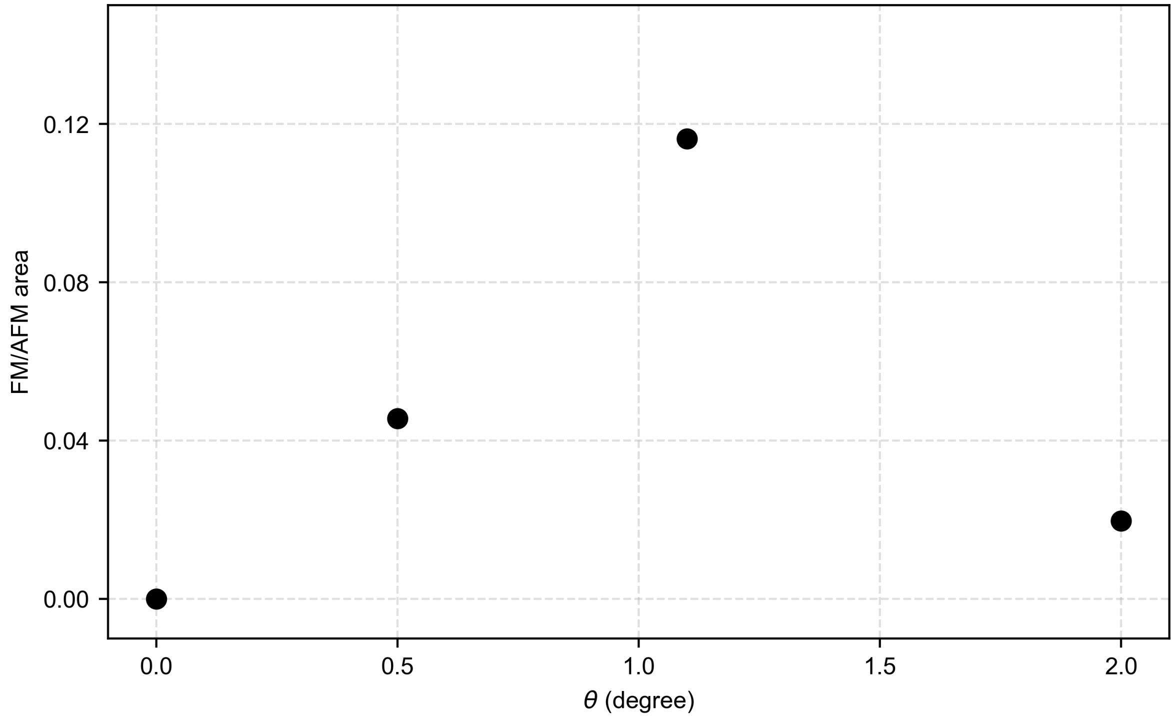

Figure S 3: FM to AFM area ratio statistics of tDB CrI_3. Each data point came from the data of one sample device. At 1.1°, this ratio is the highest, reaching around 0.12.

In addition to examining the domain wall width, we also analyzed the net magnetization of tDB CrI_3 devices at various twist angles by assessing the ratio between ferromagnetic (FM) and antiferromagnetic (AFM) regions. Our analysis indicates that the maximum net magnetization occurs at a twist angle of 1.1°, consistent with earlier RMCD measurements. However, it is important to highlight that RMCD has a spatial resolution of only a few microns, capturing the overall magnetic response rather than resolving localized magnetic features. The strong RMCD signals may be dominated by randomly distributed FM domains. In contrast, our scanning system provides more quantitative and localized insights, allowing us to resolve finer magnetic textures that are otherwise hidden in the broader RMCD measurements.

5 Autocorrelation in FM domains

5.1 Background subtraction of the FM domain in 0.5° tDB CrI_3

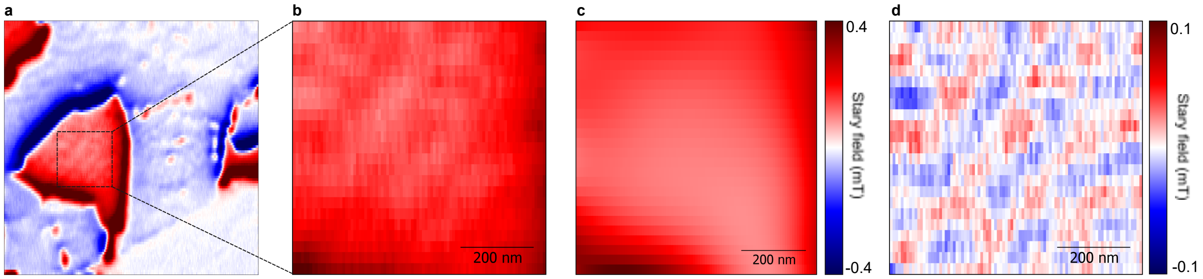

Figure S 4: **a**. The stray field map of a 0.5° tDB CrI_3 after 0.5T out-of-plane field cool down, showing randomly distributed FM and AFM domains. **b**. The enlarged map of the selected area in (a) showing a weak oscillating signal within this FM domain region. **c**. The subtracted 3rd order polynomial background in (b). **d**. Stray field map of (b) after background subtraction, revealing weak periodic feature of $\approx 20\mu$T.

5.2 Autocorrelation of the FM domain in 0.5° tDB CrI_3

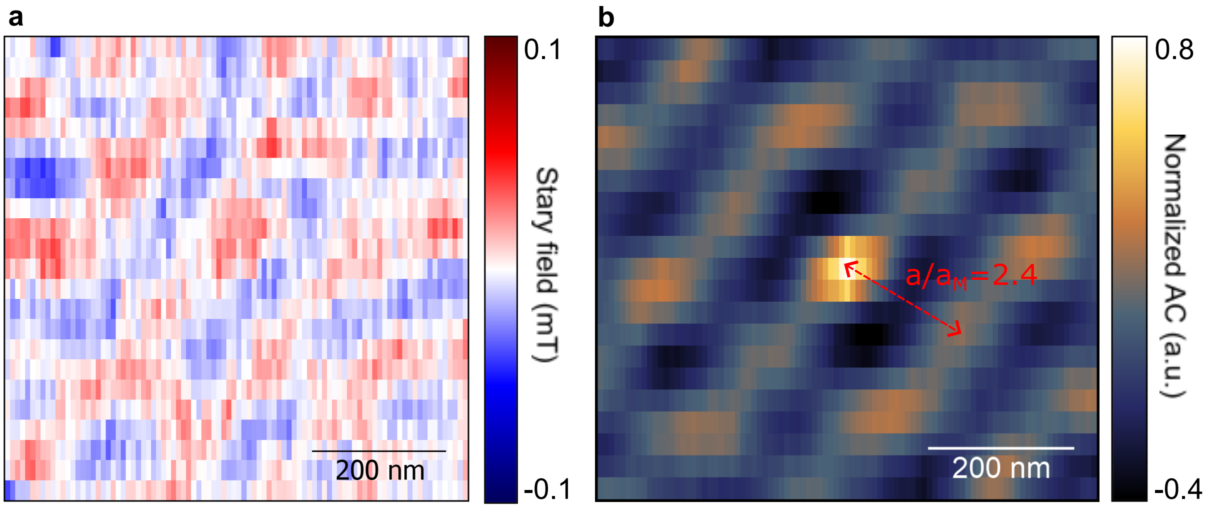

Figure S 5: **a**. The stray field map of a 0.5° tDB CrI_3 after background subtraction. **b**. The autocorrelation of (a), showing the underlying periodicity even more clearly, with a wavelength of ≈178 nm. The ratio of AC wavelength to Moire wavelength $a/a_M = 2.4$.

5.3 Background subtraction of the FM domain in 1.1° tDB CrI_3

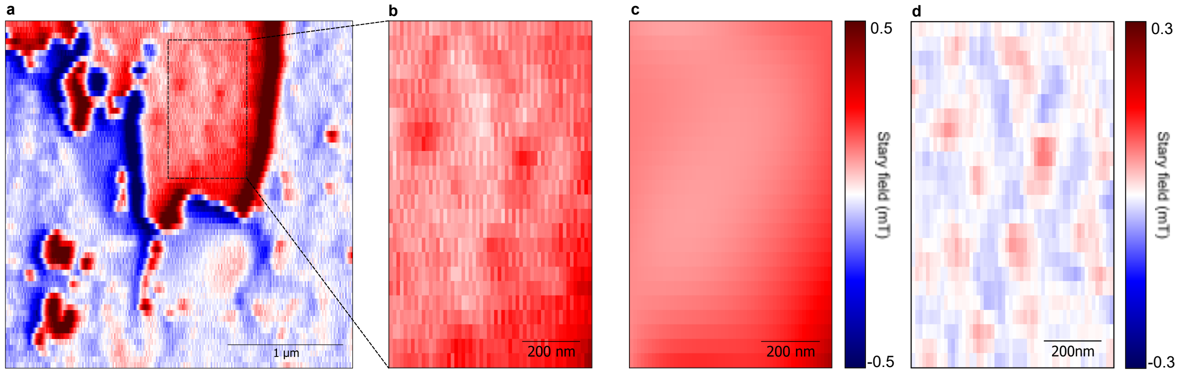

Figure S 6: **a**. The stray field map of a 1.1° tDB CrI_3 after 0.5T out-of-plane field cool down, showing randomly distributed FM and AFM domains. **b**. The enlarged map of the selected area in (a) showing a weak oscillating signal within this FM domain region. **c**. The subtracted 3rd order polynomial background in (b). **d**. Stray field map of (b) after background subtraction, revealing weak periodic feature of $\approx 20\mu$T.

5.4 Autocorrelation of the FM domain in 1.1° tDB CrI_3

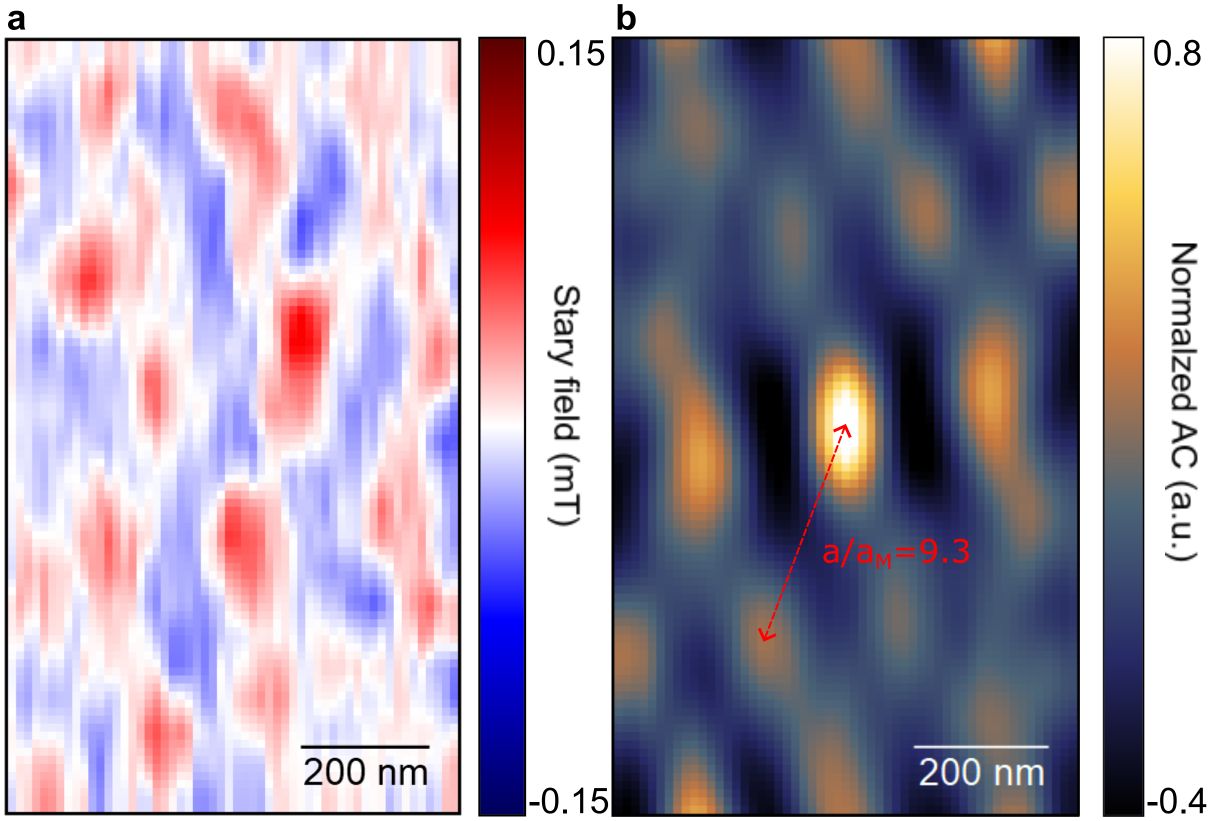

Figure S 7: **a**. The stray field map of a 1.1° tDB CrI_3 after background subtraction. **b**. The autocorrelation of (a), showing the underlying periodicity even more clearly, with a wavelength of ≈339 nm. The ratio of AC wavelength to Moire wavelength $a/a_M = 9.3$.

6 Additional autocorrelation in the AFM domains

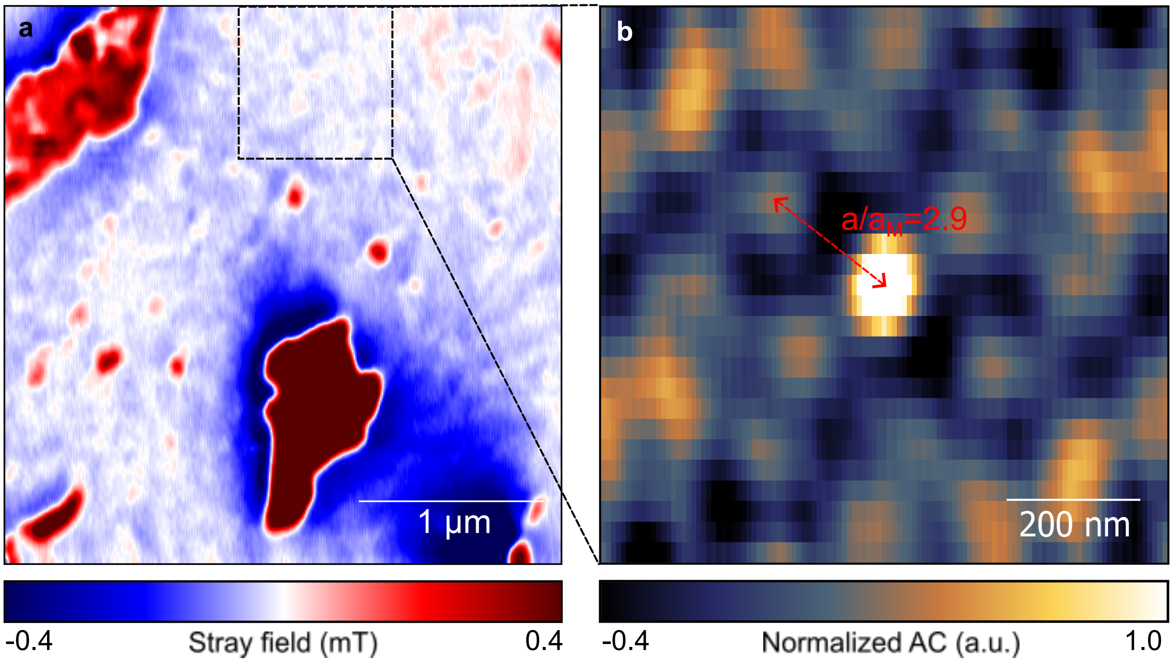

Figure S 8: **a**. Representative stray field maps of 0.5° tDB CrI_3 sample. **b**. The autocorrelation map of the selected area (black dashed box). The red dashed line indicates the periodicity in the autocorrelation, which is around 213 nm.

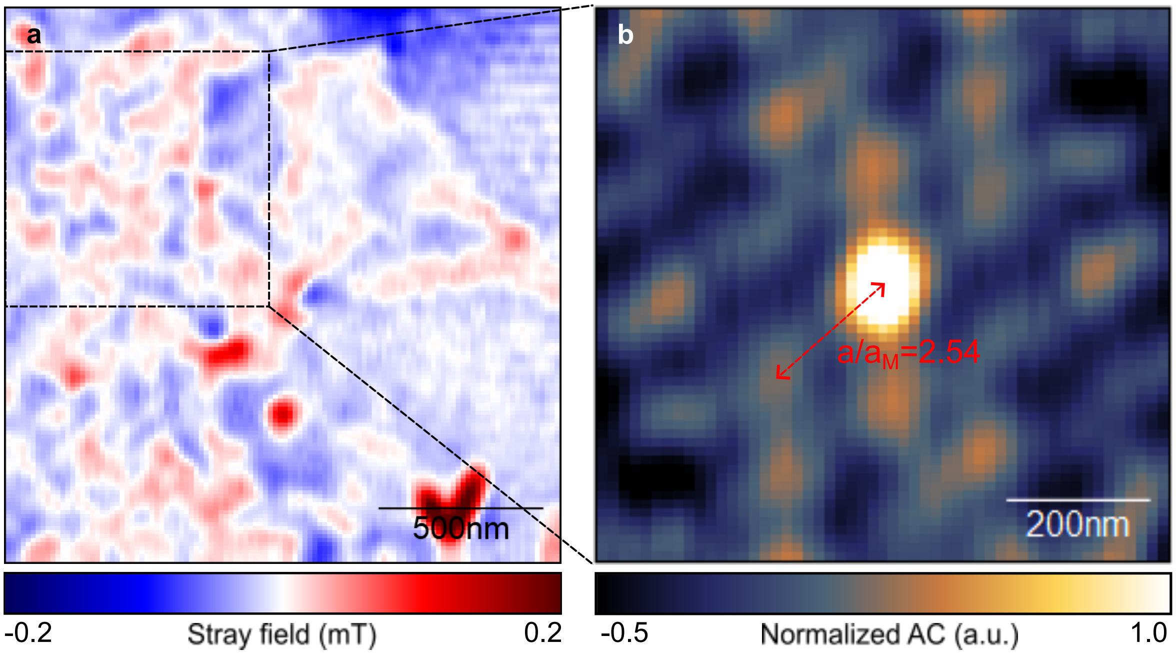

Figure S 9: **a**. Representative stray field maps of 0.5° tDB CrI_3 sample. **b**. The autocorrelation map of the selected area (black dashed box). The red dashed line indicates the periodicity in the autocorrelation, which is around 189 nm.

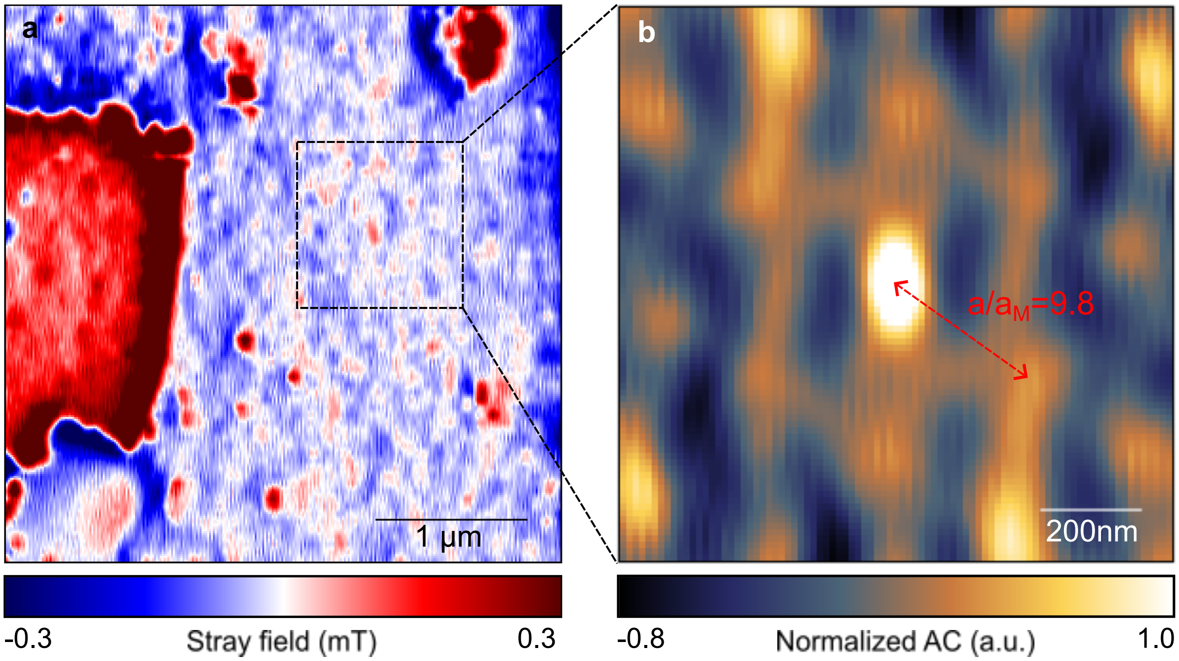

Figure S 10: **a**. Representative stray field maps of 1.1° tDB CrI_3 sample. **b**. The autocorrelation map of the selected area (black dashed box). The red dashed line indicates the periodicity in the autocorrelation, which is around 331 nm.

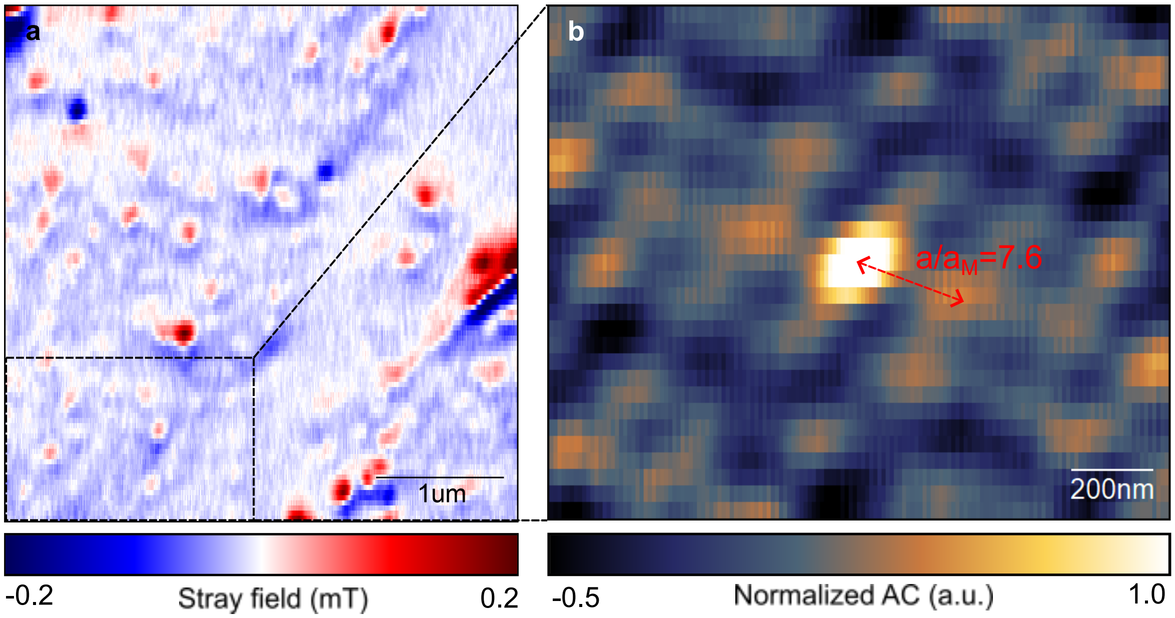

Figure S 11: **a**. Representative stray field maps of 1.1° tDB CrI_3 sample. **b**. The autocorrelation map of the selected area (black dashed box). The red dashed line indicates the periodicity in the autocorrelation, which is around 258 nm.

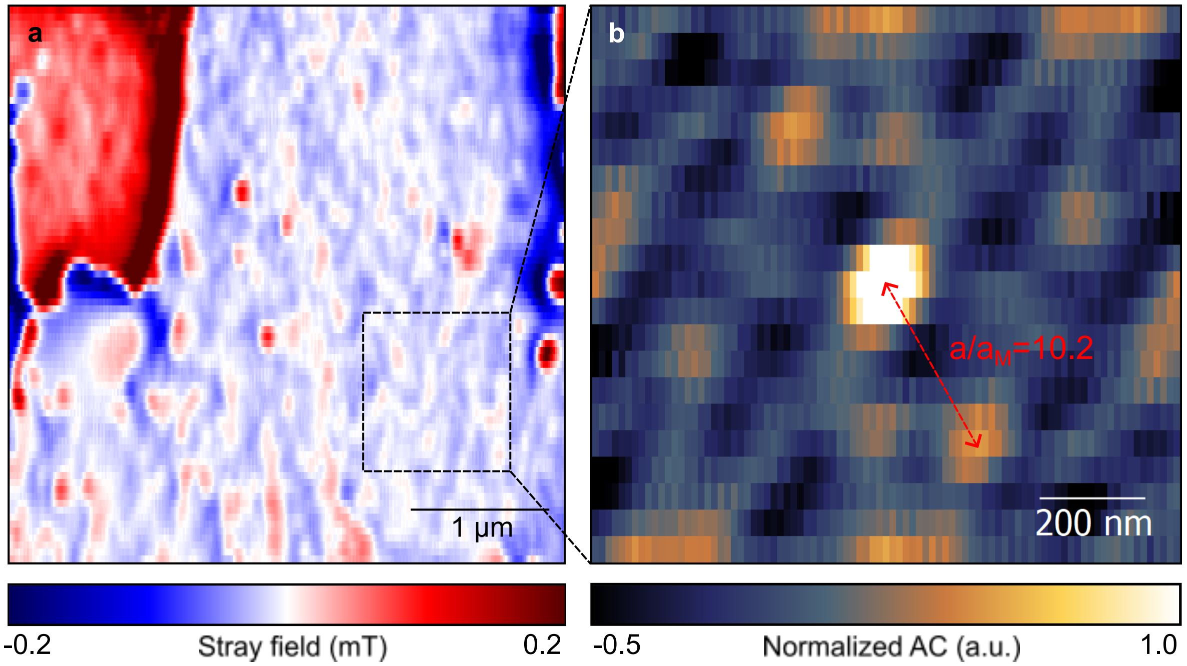

Figure S 12: **a**. Representative stray field maps of 1.1° tDB CrI_3 sample. **b**. The autocorrelation map of the selected area (black dashed box). The red dashed line indicates the periodicity in the autocorrelation, which is around 346 nm.

7 Magnetic field dependence measurement of AFM domain

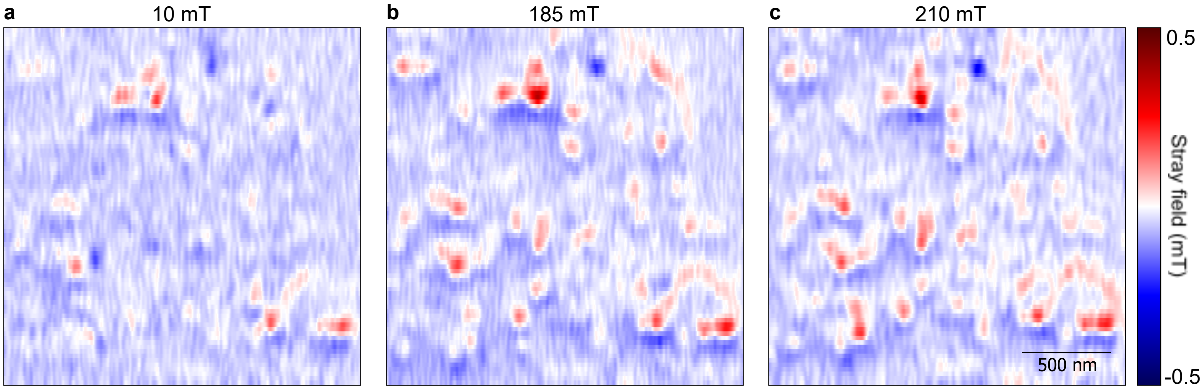

Figure S 13: **a-c**. Representative stray field maps of a 1.1° tDB CrI_3 sample after 500 mT field cooldown, measured with a bias field of 10 mT, 185 mT and 210 mT parallel to the NV axis, respectively. One can observe these dot-like features becoming more prominent as the field increases from 10 mT to 210 mT.

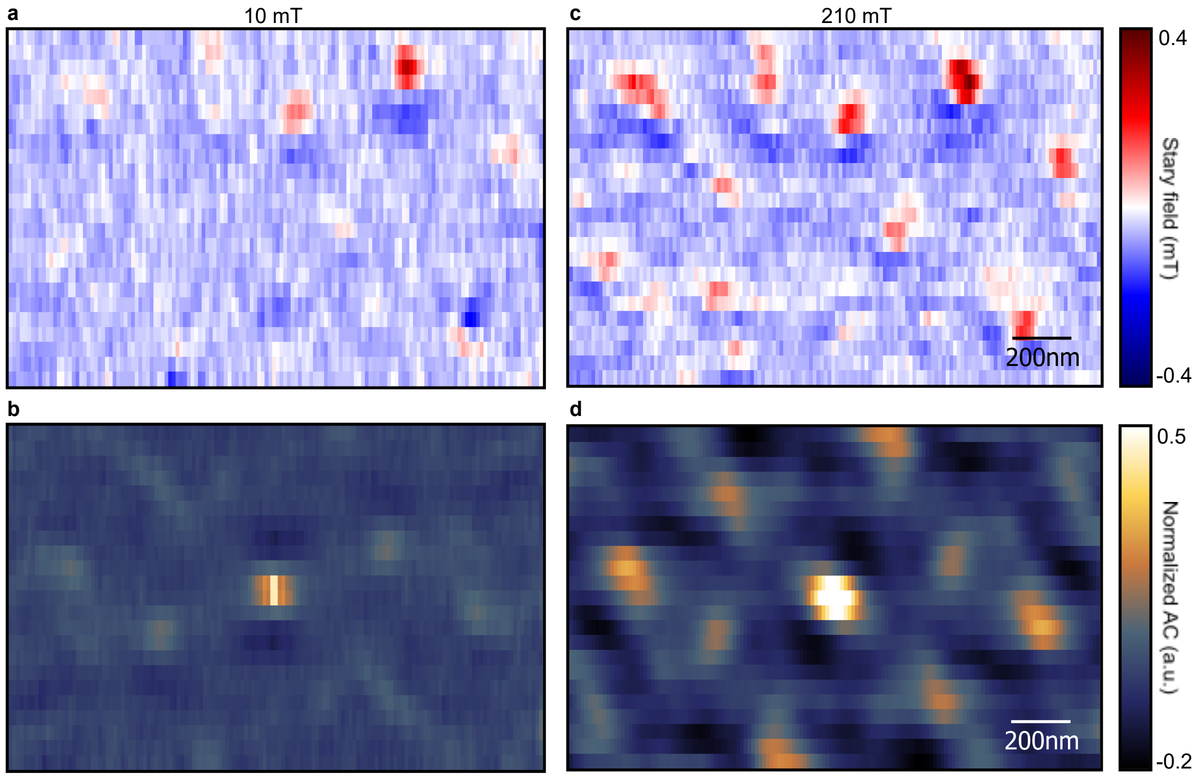

Figure S 14: **a**. A representative stray field map of a 1.1° tDB CrI_3 sample after 500 mT field cooldown measured at 10 mT parallel to the NV axis, dot-like features are visualized in the AFM region. **b** The autocorrelation of (a). **c**. The stray field map of the same area at 210 mT parallel to the NV axis, the dot-like features became more prominent. **d**. The autocorrelation of (c). The resonance peaks observed in (b) became more prominent.

8 The Stray field map at the edge of 0.5° tDB CrI_3

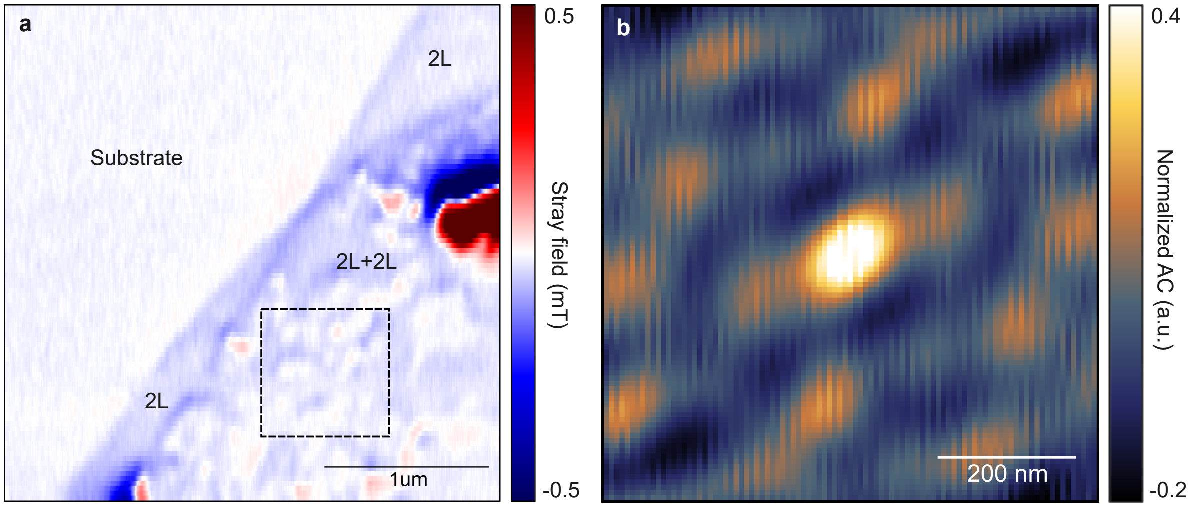

Figure S 15: **a**. The stray field map at the edge of a tDB CrI3 device. Substrate, pristine 2L, and twisted double bilayer (tDB) regions are visible. FM domains and magnetic textures are observed only in the twisted region. **b**. The autocorrelation of a selected area in (a).

9 Pristine 4L and 2L CrI_3 devices

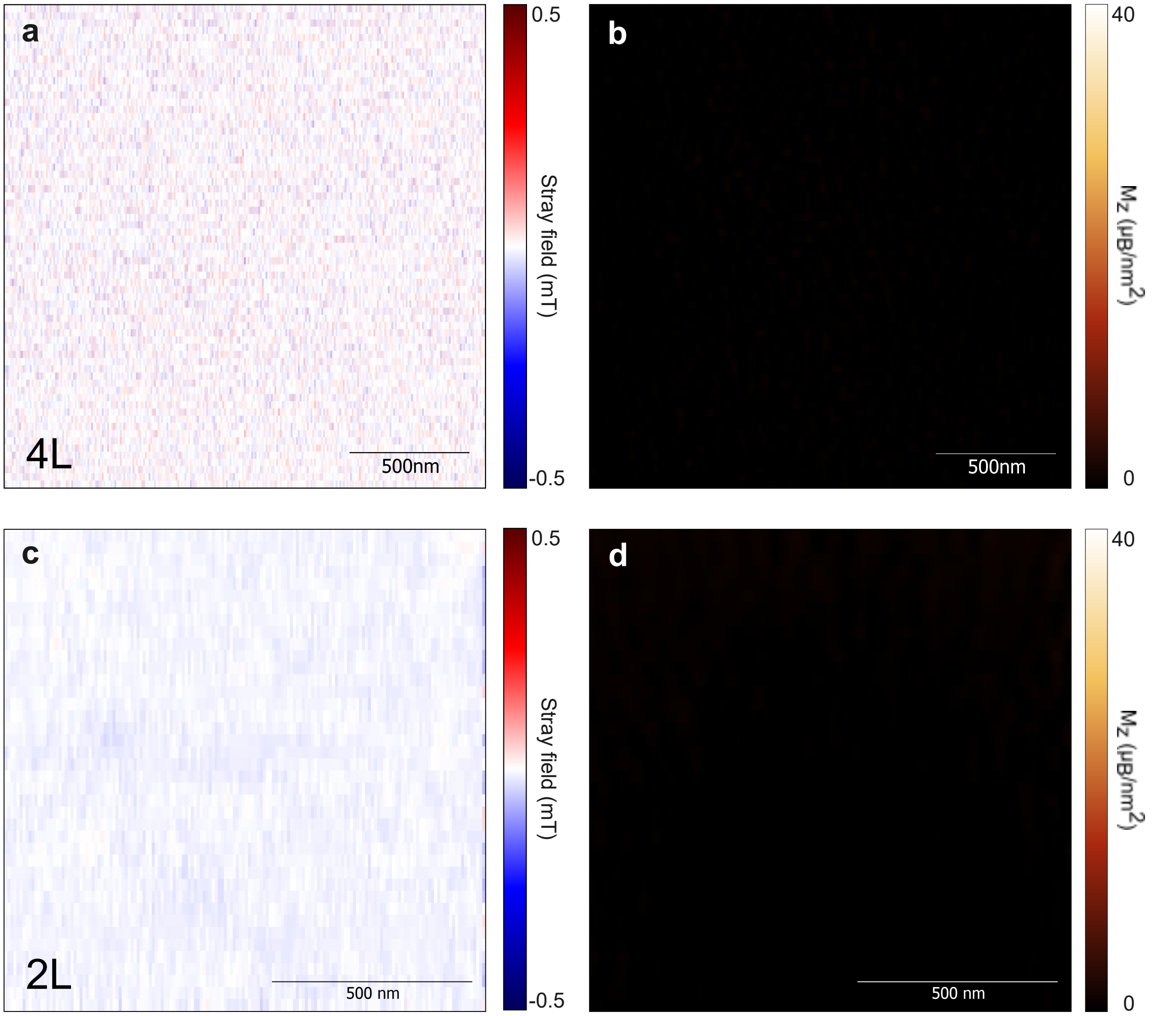

Figure S 16: **a**. The stray field map obtained from NV-AFM for pristine 4L of CrI_3, showing uniform and nearly zero field strength across the region. **b**. The reconstructed M_z map computed from (a), showing nearly zero magnetization. **c**. The stray field map for a pristine 2L of CrI_3, also showing uniform and nearly zero field strength across the region. **d**. The reconstructed M_z map computed from (c).

10 Stray field maps of 1.2° tDT and 2.0° tDB CrI_3

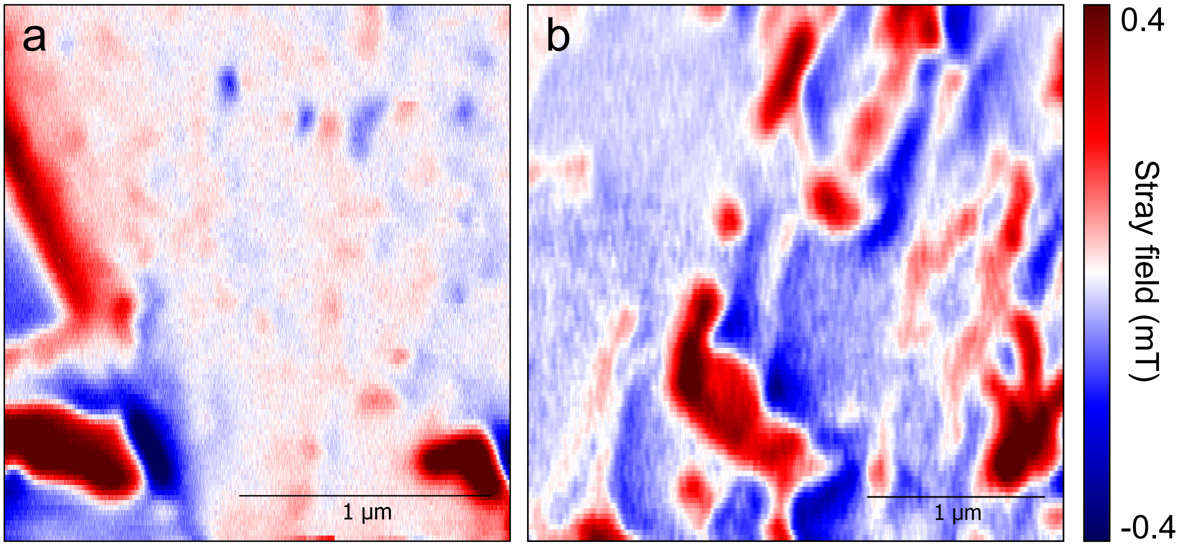

Figure S 17: **a**. Representative stray field map of a 1.2° twisted double trilayer CrI_3 sample after 500 mT field cooldown, measured with a bias field of 210 mT. In the 3L+3L region, randomly distributed FM and AFM domains are observed. **b**. Representative stray field map of a 2.0° twisted double bilayer CrI_3, showing randomly distributed FM and AFM domains. However, no periodic pattern is observed.

11 Histogram of magnetization in tDB CrI_3

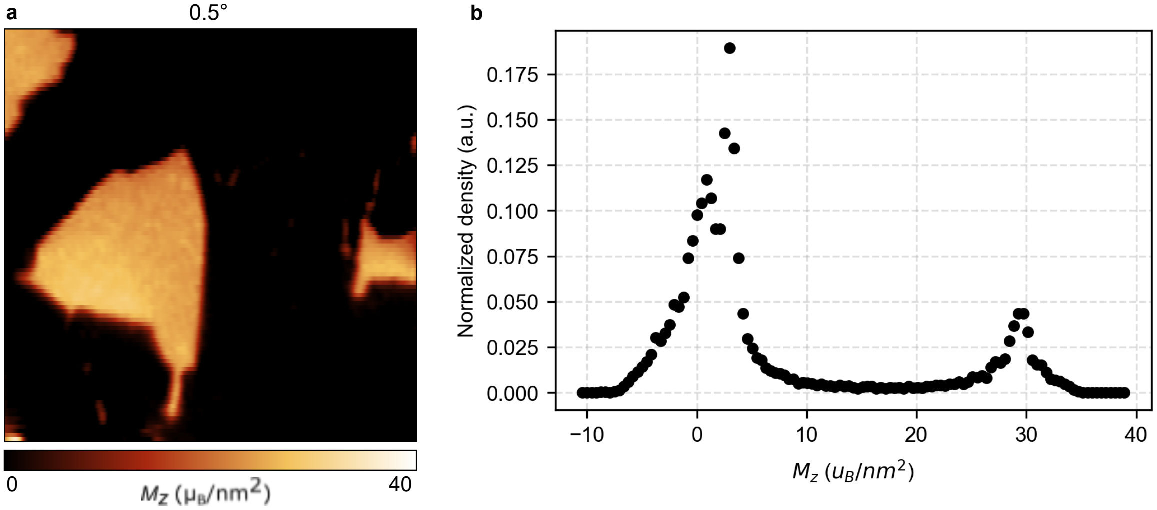

Figure S 18: **a**. A representative M_z map of a 0.5° tDB CrI_3. **b**. The M_z histogram of (a).

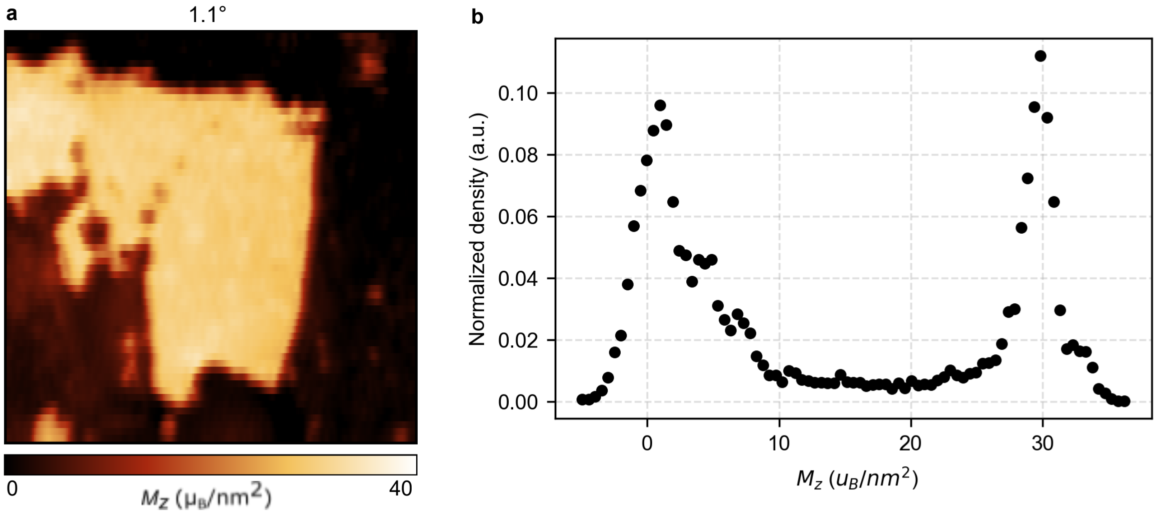

Figure S 19: **a**. A representative M_z map of a 1.1° tDB CrI_3. **b**. The M_z histogram of (a).

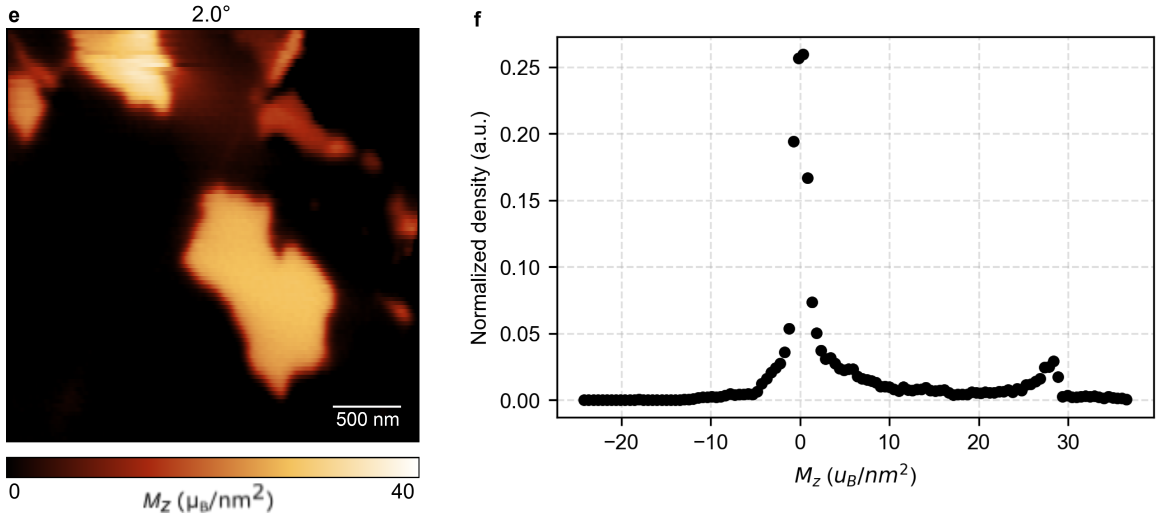

Figure S 20: **a**. A representative M_z map of a 2.0° tDB CrI_3. **b**. The M_z histogram of (a).

12 Additional magnetic field profile of the dot-like feature

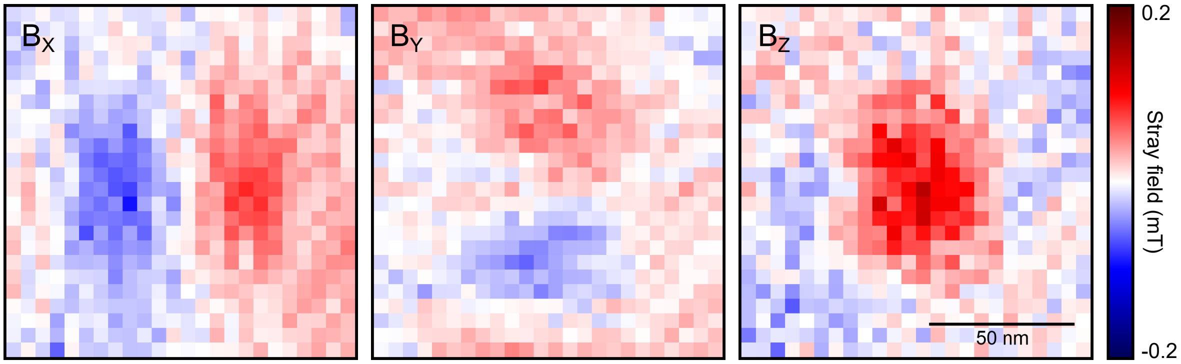

Figure S 21: B_x, B_y and B_z profile computed by reconstructing from a B_{NV} scan of the dot-like feature in 1.1° tDB CrI_3. The profile resembles a Neél-type skyrmion.

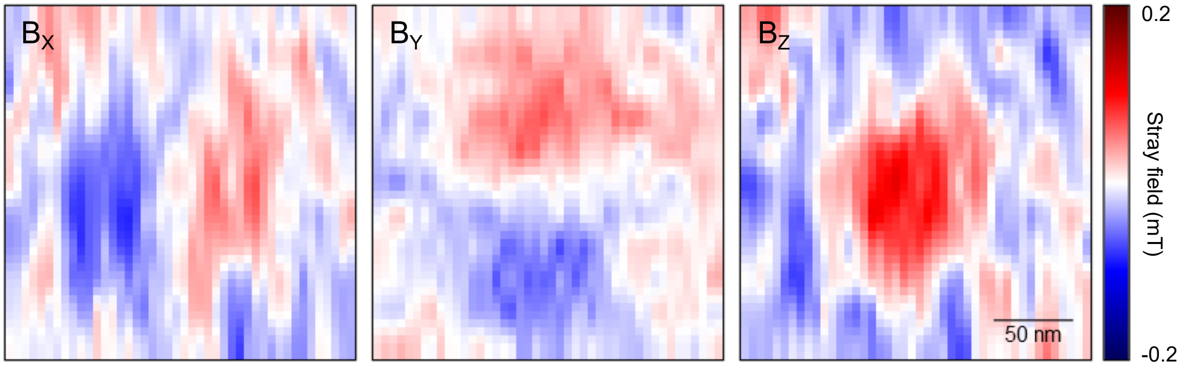

Figure S 22: B_x, B_y and B_z profile computed by reconstructing from a scan of B_{NV} on the dot-like feature in 1.1° tDB CrI_3.

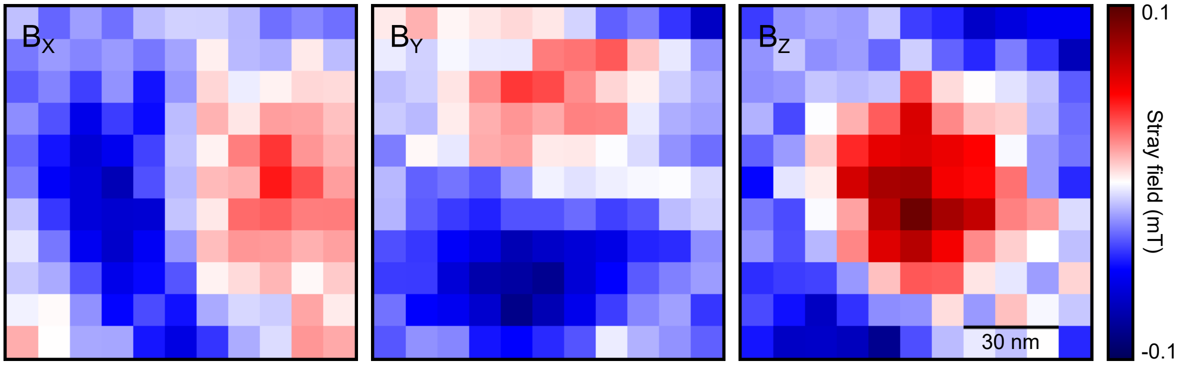

Figure S 23: B_x, B_y and B_z profile computed by reconstructing from a scan of B_{NV} on the dot-like feature in 1.1° tDB CrI_3.

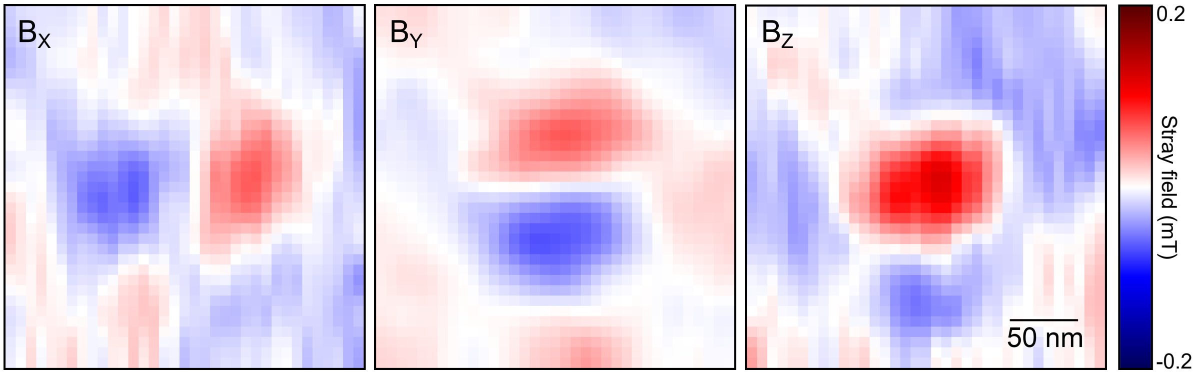

Figure S 24: B_x, B_y and B_z profile computed by reconstructing from a scan of B_{NV} on the dot-like feature in 0.5° tDB CrI_3.

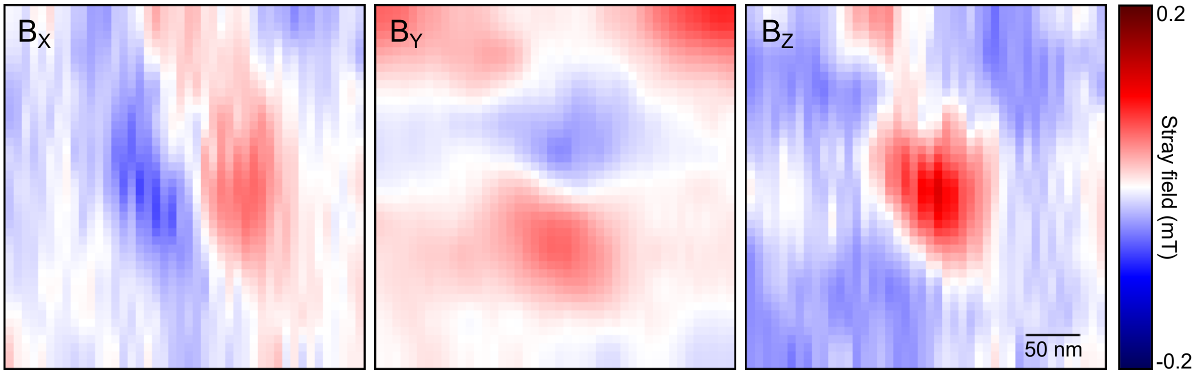

Figure S 25: B_x, B_y and B_z profile computed by reconstructing from a scan of B_{NV} on the dot-like feature in 0.5° tDB CrI_3.

13 Short summary of the magnetic features observed in all tDB CrI_3 samples

Features \ Twist angle	0°	0.5°	1.1°	2.0°
FM domains	No	Yes	Yes	Yes
AFM domains	Yes	Yes	Yes	Yes
Super Moire textures	No	Yes	Yes	No

Supplementary Table 1: Summary of magnetic features observed in tDB CrI_3 samples. For twist angles of 0.5° and 1.1°, a total of six samples (3 for each angle) were measured. Magnetic features were consistently observed across samples from two independent groups, indicating robust and reproducible behavior. For a twist angle of 2.0°, two samples were examined, and no super-Moiré magnetic features were detected.

14 Determination of sample to NV distance

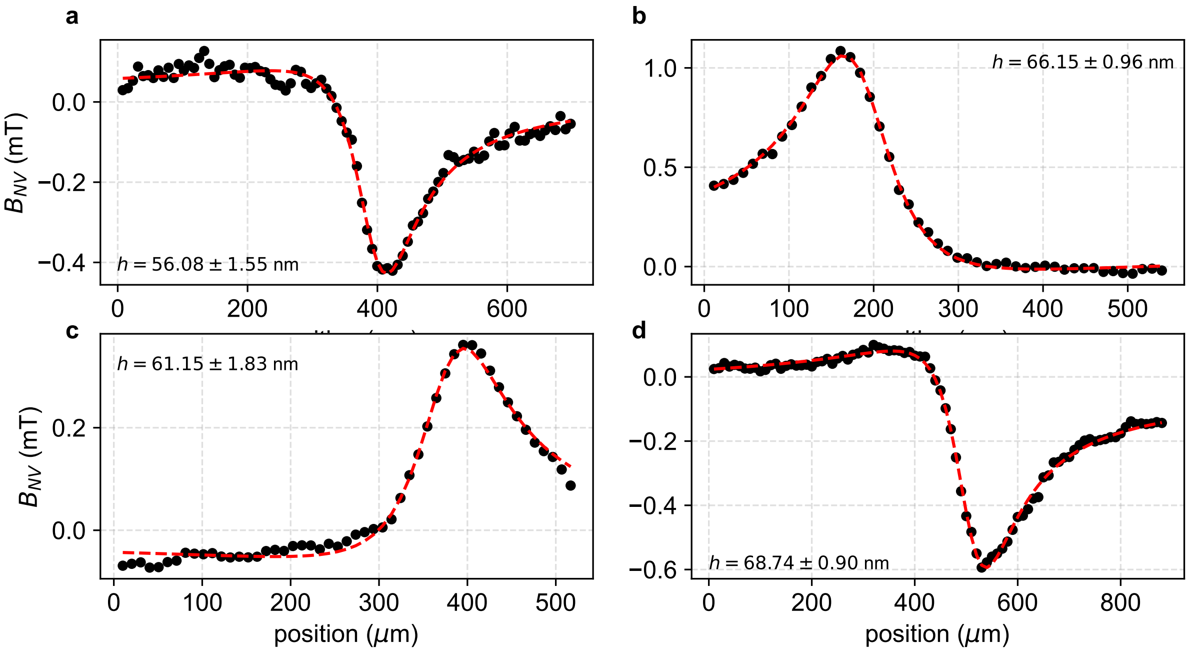

Figure S 26: **a**. B_{NV} profile across a sample edge in 0.2° tB CrI_3 device. **b**. B_{NV} profile across a sample edge in 0.5° tDB CrI_3 device. **c**. B_{NV} profile across a sample edge in 0.5° tDB CrI_3 device. **d**. B_{NV} profile across a sample edge in 1.1° tDB CrI_3 device.

15 Optical and AFM images of the tDB CrI_3 devices

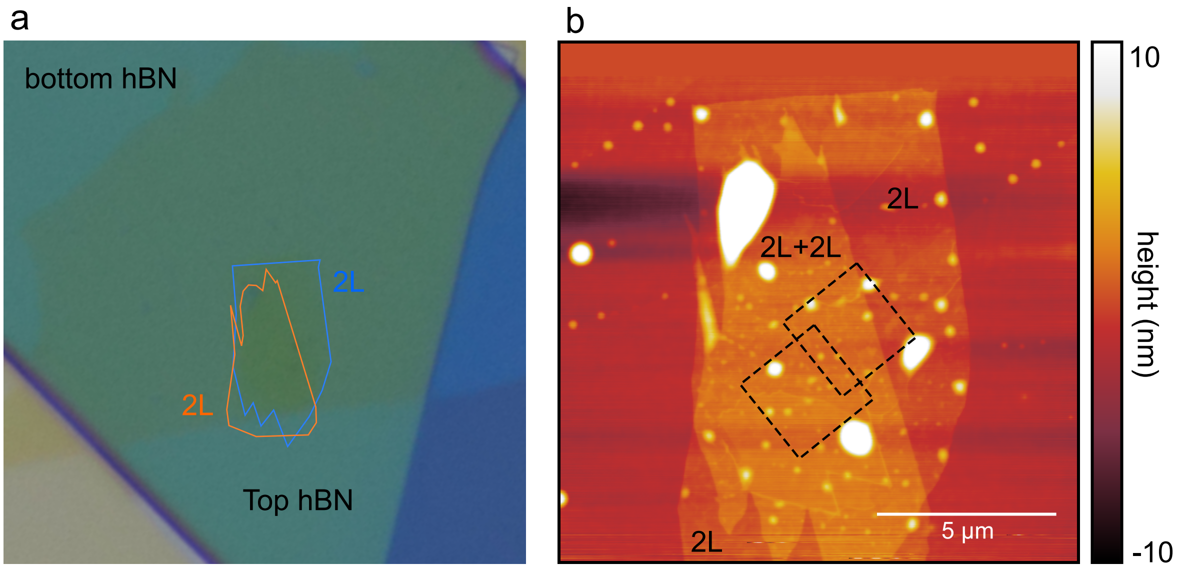

Figure S 27: **a**. Optical image of the tDB CrI_3 device of the main Fig. 2a and Fig. 3a,c. The two bilayer CrI_3 flakes are highlight in blue and orange. The hBN layers could also be seen in the image. **b**. The AFM image of the entire device using a conventional AFM. The twisted region are labeled. The black squares indicates roughly the scanning area of main Fig. 2a and Fig. 3a,c.

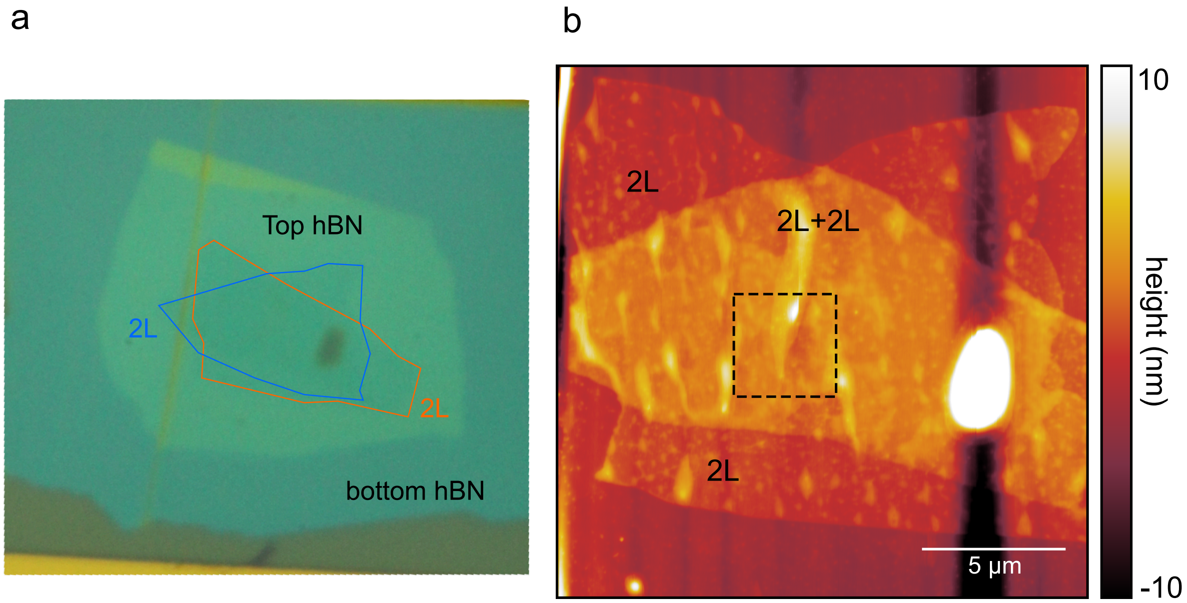

Figure S 28: **a**. Optical image of the tDB CrI_3 device of the main Fig. 2b. The two bilayer CrI_3 flakes are highlight in blue and orange. The hBN layers could also be seen in the image. **b**. The AFM image of the entire device using a conventional AFM. The twisted region are labeled. The black square indicates roughly the scanning area of main Fig. 2b.

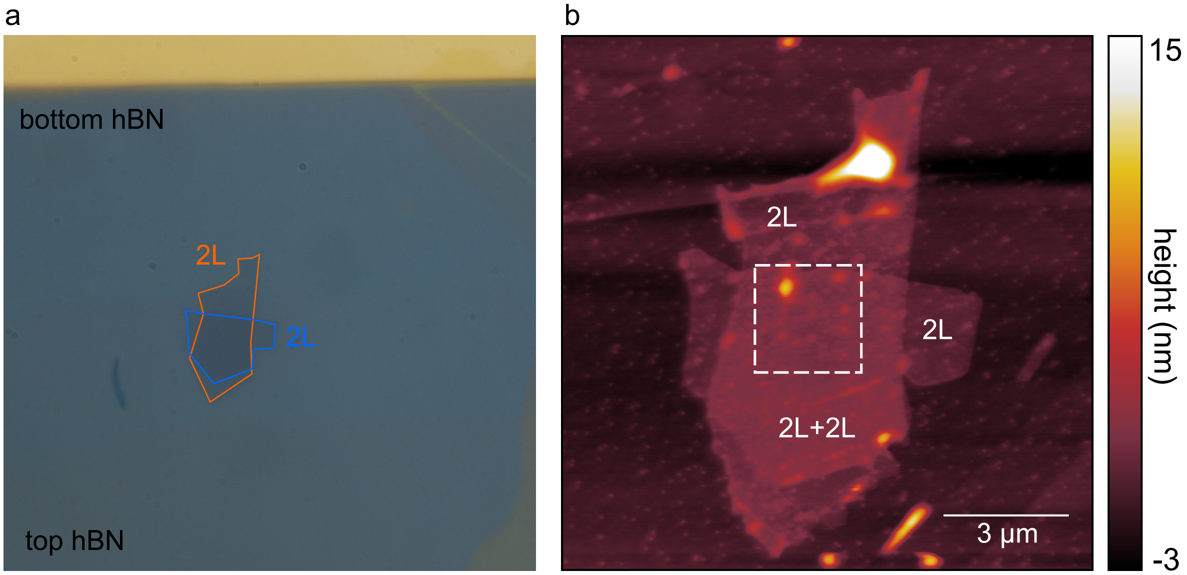

Figure S 29: **a**. Optical image of the tDB CrI_3 device used in the SI. The two bilayer CrI_3 flakes are highlight in orange and blue with an indicator. The hBN layers could also be seen in the image. **b**. The AFM image of the entire device using a conventional AFM. The twisted region are labeled. The white square indicates roughly the scanning area of SI Fig. S39.

16 Optical images of a 1.1° tDB CrI_3 devices and the RMCD measurement

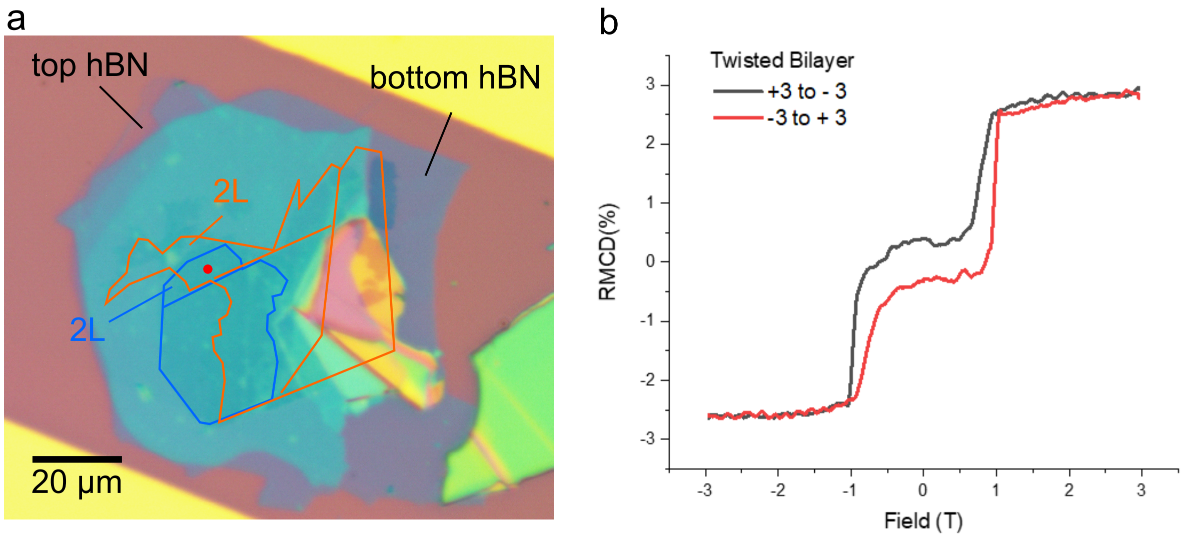

Figure S 30: **a**. Optical image of the tDB CrI_3 device of the main Fig. 3e. The two bilayer CrI_3 flakes are highlight in blue and orange. The hBN layers could also be seen in the image. The red indicator represent the RMCD measurement spot. **b**. The RMCD versus external field sweep of the tDB CrI_3 area, showing similar quality as previous reported.

17 AFM images of the hBN flakes used to encapsulate sample devices

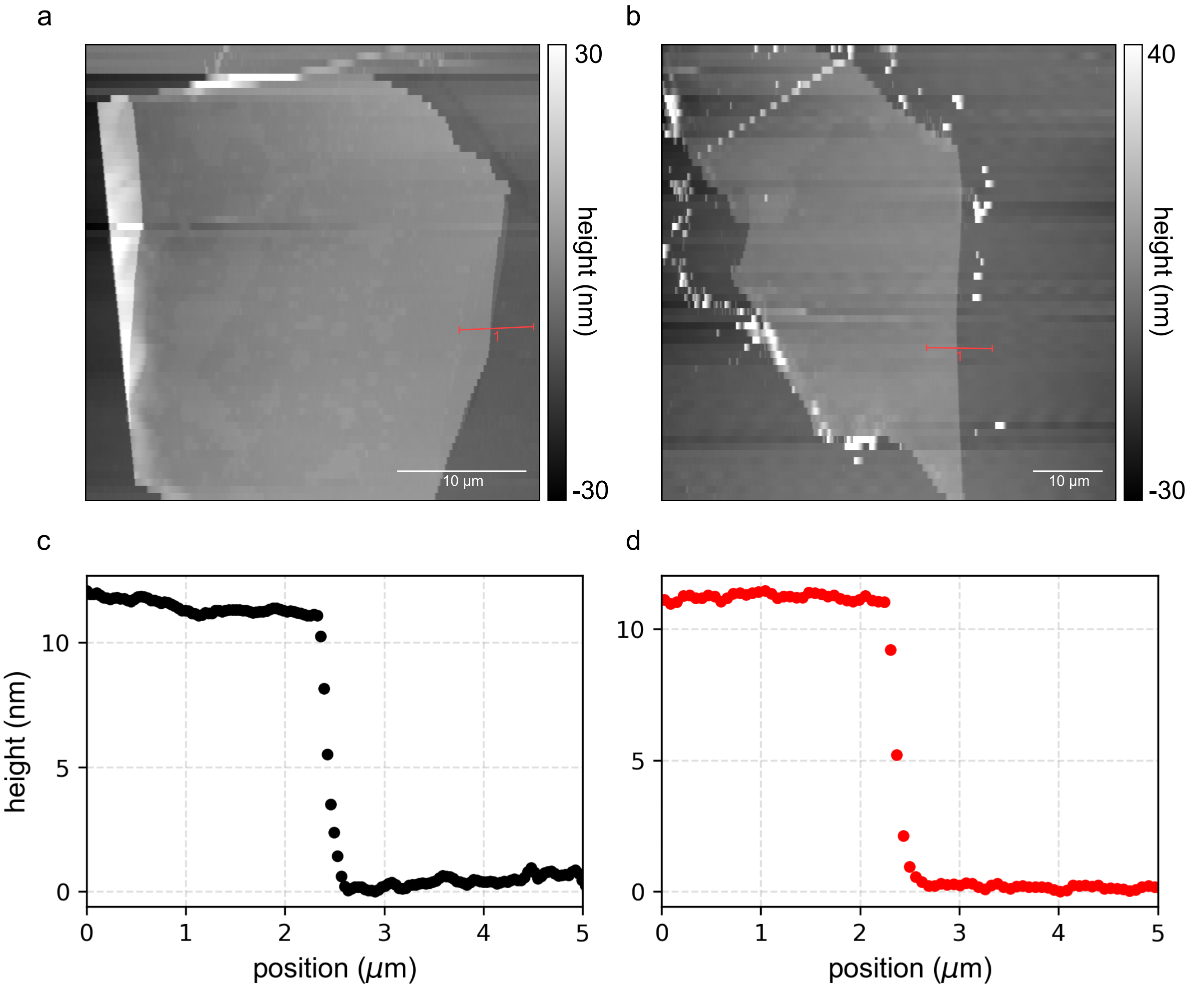

Figure S 31: **a,b**. AFM images of the hBN flake used for the sample device in main Fig.2a, 2b, 3a and 3c. **c,d**. Linecuts made on the AFM maps in (a) and (b) showing hBN thickness of about 11nm. The linecut is marked in red in the AFM images.

18 Stray field map and topography comparison 01

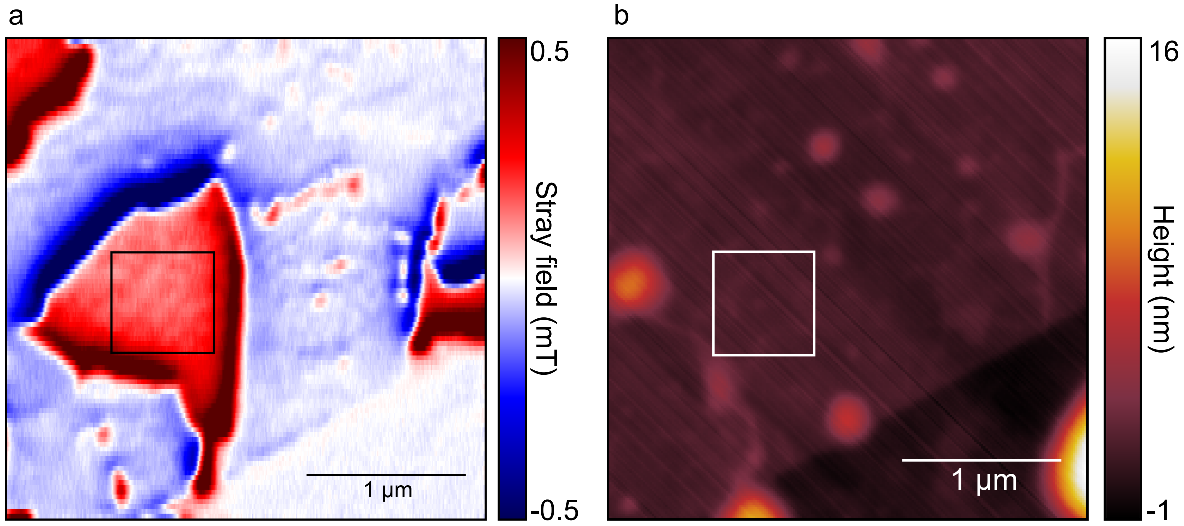

Figure S 32: **a**. Stray field map of the main Fig. 2a. The black square highlight the area for autocorrelation. **b**. AFM images of the same scanning area. White square highlight the area for autocorrelation.

Stray field map and topography comparison 02

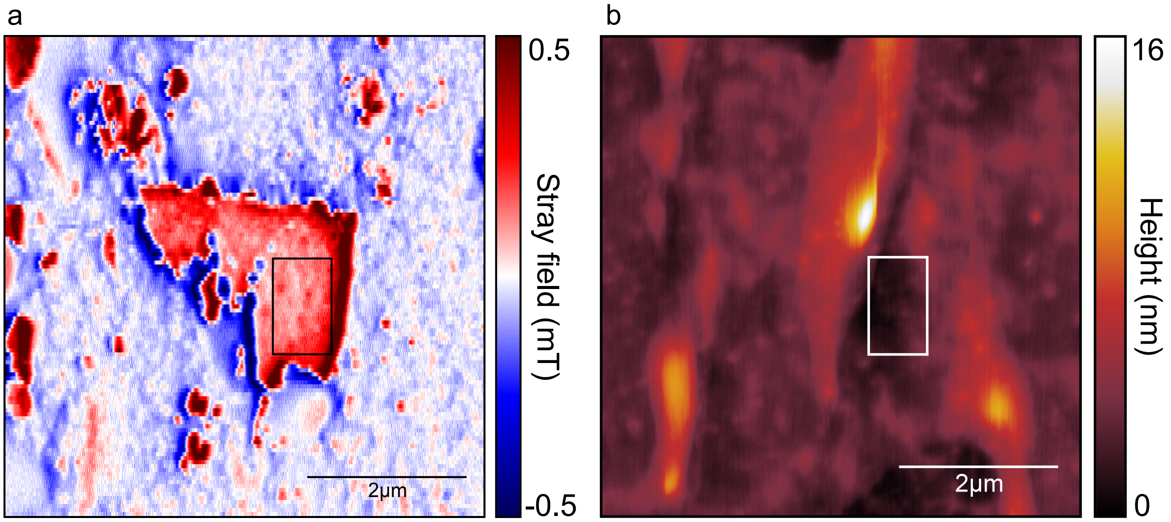

Figure S 33: **a**. Enlarged stray field map of the main Fig. 2b. The black box highlight the area for autocorrelation. **b**. AFM images of the same scanning area. White box highlight the area for autocorrelation.

Stray field map and topography comparison 03

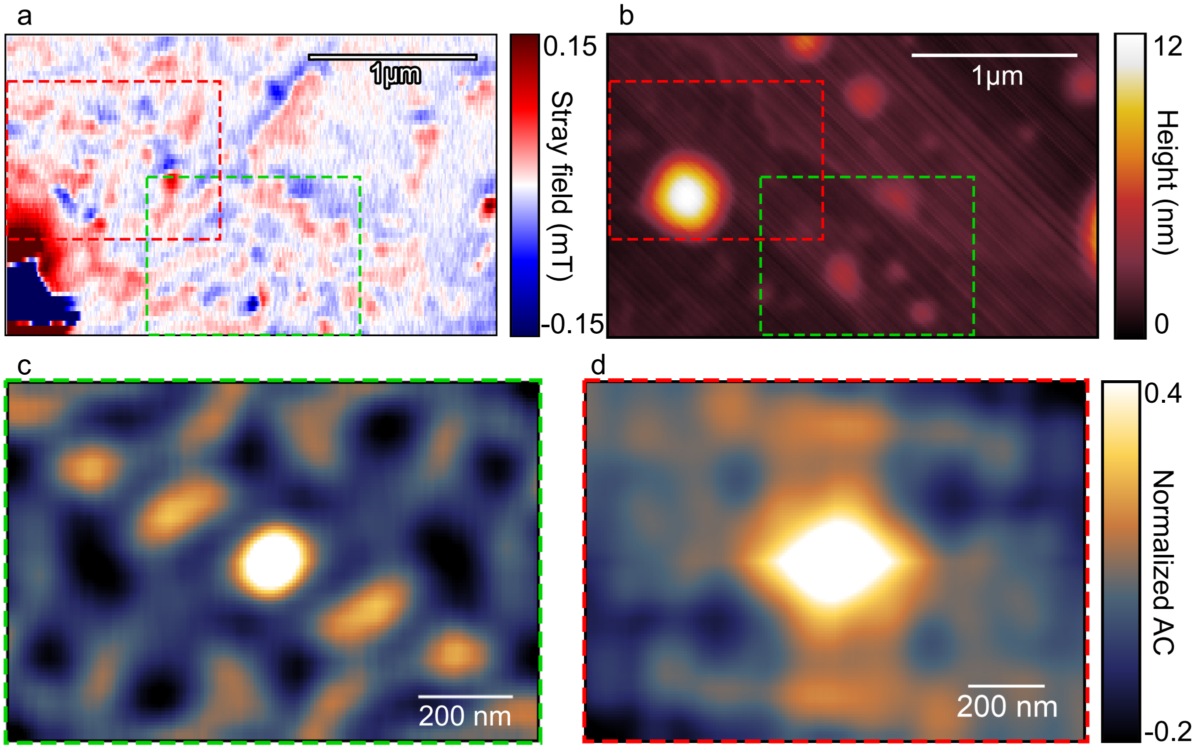

Figure S 34: **a**. Stray field map of the main Fig. 3a. The green and red dashed boxes indicated a relatively flat and rough area, respectively. **b**. The AFM image of the same scanning area. Green and red dashed boxes highlight the corresponding areas. **c**. Autocorrelation of the green area. **d**. Autocorrelation of the red area.

Stray field map and topography comparison 04

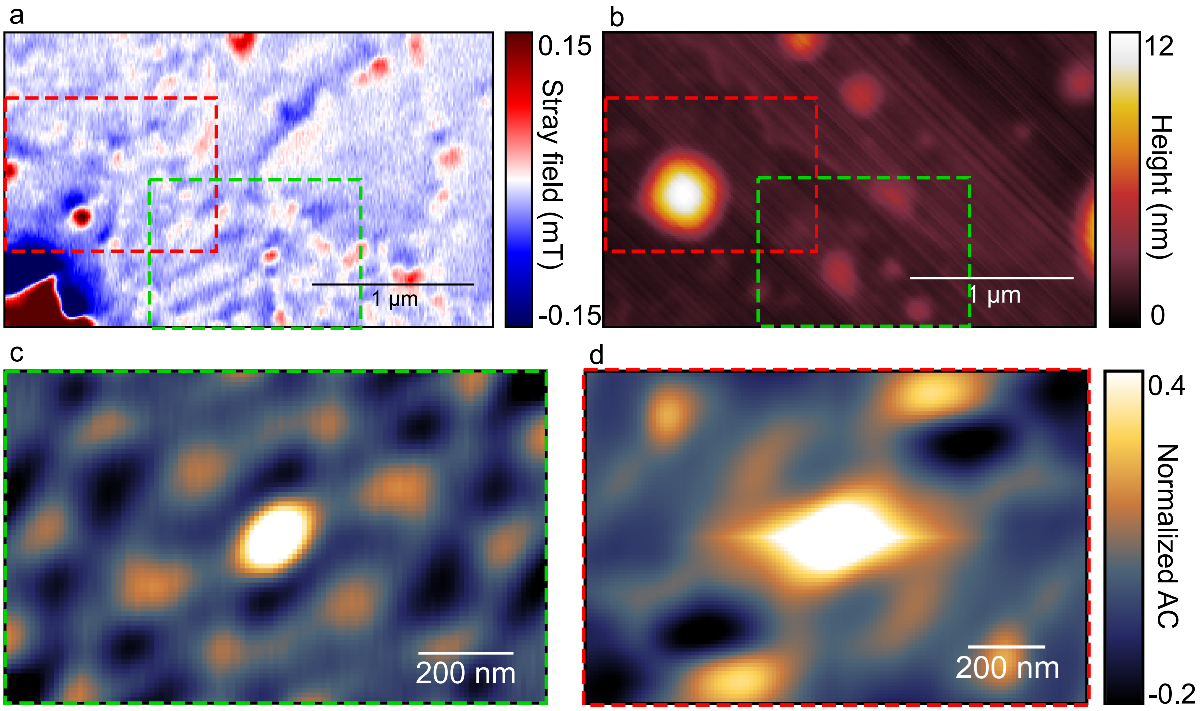

Figure S 35: **a**. Stray field map of the main Fig. 3c. The green and red dashed boxes indicated a relatively flat and rough area, respectively. **b**. The AFM image of the same scanning area. Green and red dashed boxes highlight the corresponding areas. **c**. Autocorrelation of the green area. **d**. Autocorrelation of the red area.

Stray field map and topography comparison 05

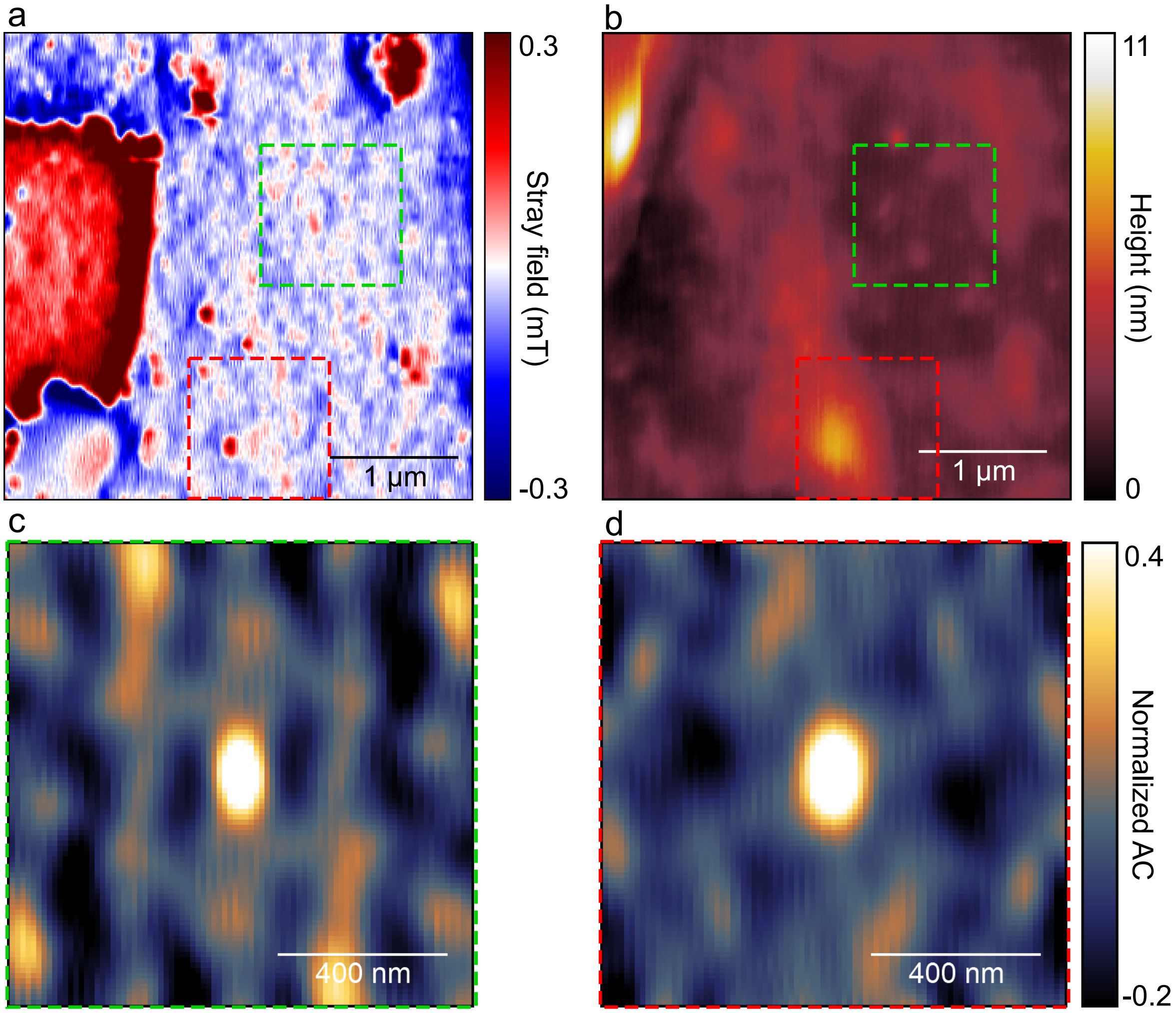

Figure S 36: **a**. Stray field map of Fig. S10. The green and red dashed boxes indicated a relatively flat and rough area, respectively. **b**. The AFM image of the same scanning area. Green and red dashed boxes highlight the corresponding areas. **c**. Autocorrelation of the green area. **d**. Autocorrelation of the red area.

Stray field map and topography comparison 06

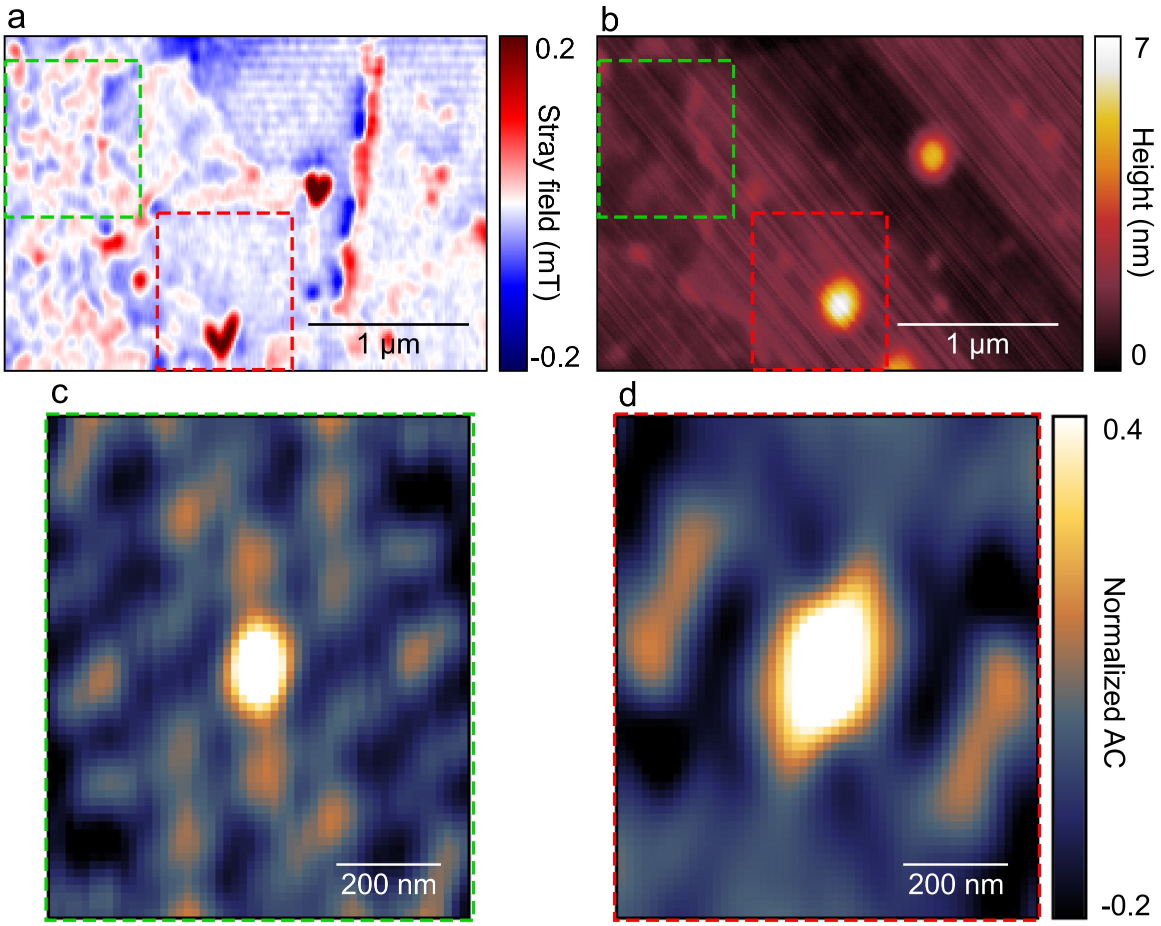

Figure S 37: **a**. Stray field map of Fig. S9. The green and red dashed boxes indicated a relatively flat and rough area, respectively. **b**. The AFM image of the same scanning area. Green and red dashed boxes highlight the corresponding areas. **c**. Autocorrelation of the green area. **d**. Autocorrelation of the red area.

Stray field map and topography comparison 07

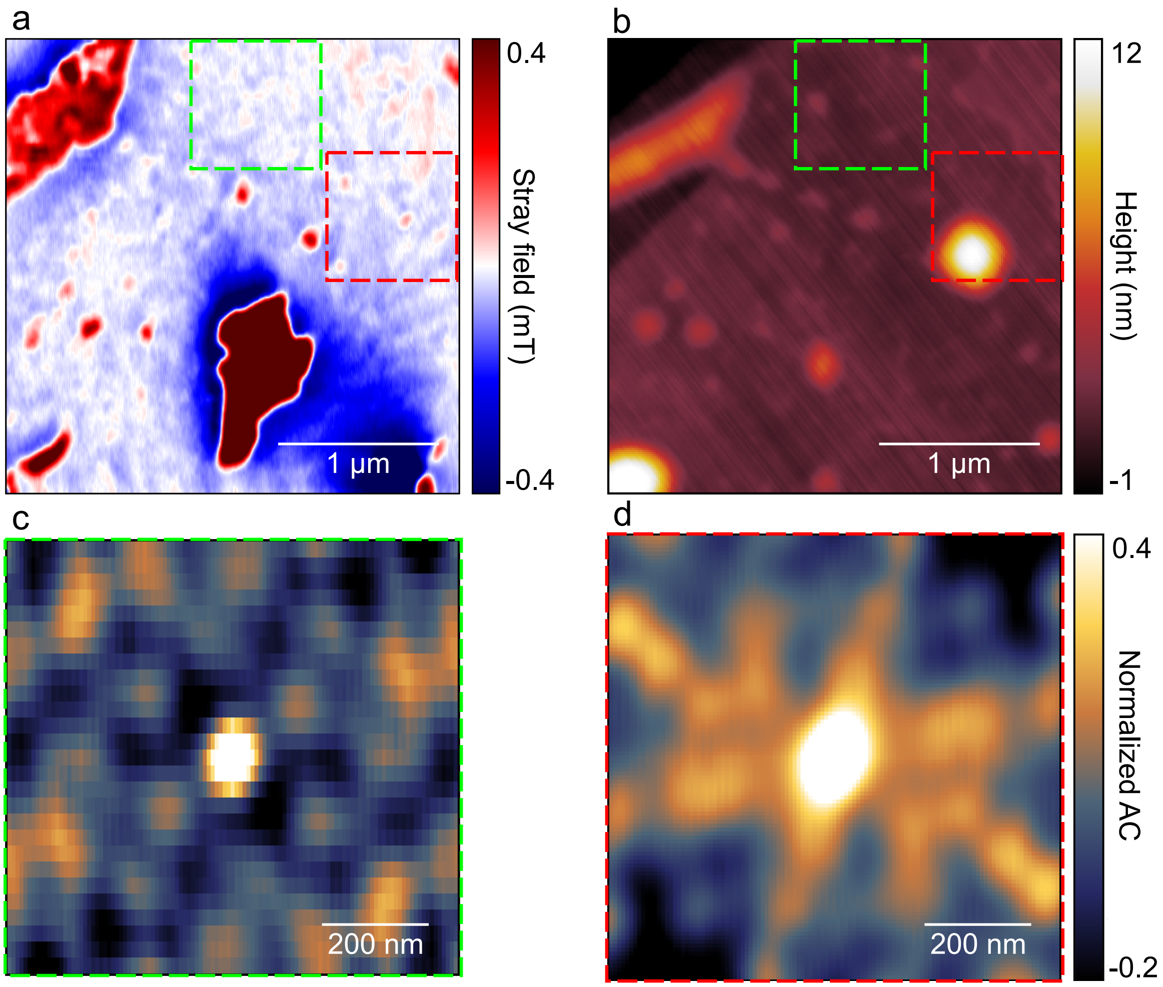

Figure S 38: **a**. Stray field map of Fig. S8. The green and red dashed boxes indicated a relatively flat and rough area, respectively. **b**. The AFM image of the same scanning area. Green and red dashed boxes highlight the corresponding areas. **c**. Autocorrelation of the green area. **d**. Autocorrelation of the red area.

Stray field map and topography comparison 08

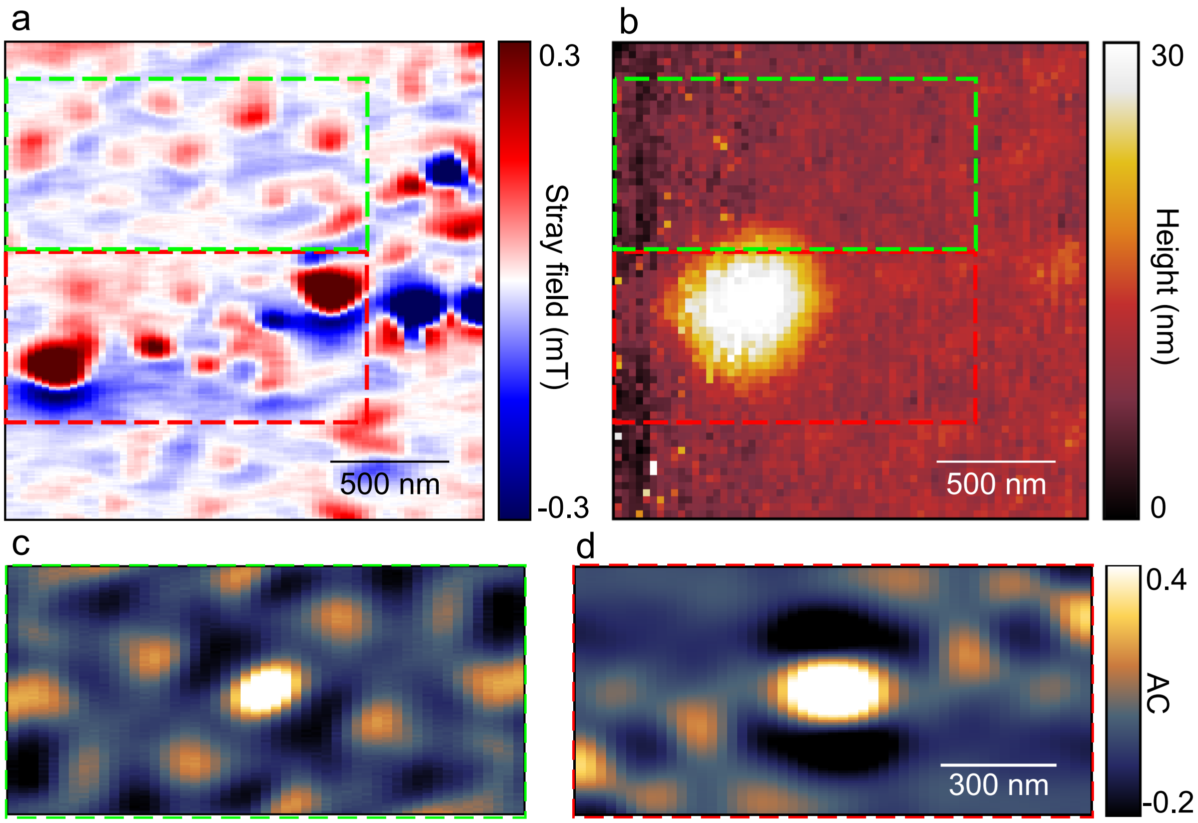

Figure S 39: **a**. Stray field map of a 0.5° tDB CrI_3 device. The green and red dashed boxes indicated a relatively flat and rough area, respectively. **b**. The AFM image of the same scanning area. Green and red dashed boxes highlight the corresponding areas. **c**. Autocorrelation of the green area. **d**. Autocorrelation of the red area.

19 Simulation of tDB CrI_3

19.1 Method

The simulated spin configurations were obtained using the atomistic modeling code VAMPIRE (*3*) using an an adaptive solver (*4*) rather than a simple random-step algorithm. This allows optimal phase-space sampling on the trial move of the spins keeping the acceptance rate close to 50% (Metropolis golden rule), which enhances efficiency and generates low correlation times. This makes convergence to the global ground state significantly faster than in traditional brute-force MC algorithms, especially minimizing the lifetime of metastable states, which is a common difficulty for MC simulations in general. The ground state of each system was determined from initial random spin orientations evolved at various temperatures until convergence was achieved. We used a large number of MC-steps (400,000−600,000 steps) at 0 T, 0.21 T, and 0.51 T field cooling to ensure full convergence of the global ground state. System size ranges from 1,000,000 to 10,000,000 atomic spin sites, which were fully converged before analysis. Computing resources for the MC simulations range from 10-20 nodes with 100-500 cores total using 1 Gb per core for ∼20-35 hours. Note the used memory is the running memory, which is more optimized than the setup memory. The setup memory — even after optimizations — still requires ≈30 Gb per core.

Unless otherwise stated, the simulations occur at 4.2 K with a system involving four CrI_3 layers in their respective stacking order (*5, 6*) at the size of 400 nm $\times$ 400 nm for each layer. We model the atomic magnetic moments using the spin Hamiltonian containing terms for the isotropic bilinear exchange, DMI exchange, and single ion anisotropy (Eq. 22). To make a prompt analysis of the systems, the exchange terms are characterized with an isotropic component J and an anisotropic component D with symmetry governed through DMI. J_{ij}^{lm} describes the isotropic exchange between spins i and j in layers l and m. $\mathbf{D}_{ij}^{lm}$ is the DMI exchange vector

between spins i and j. Terms with layers $l = m$ correspond to intra-layer interactions. $\mathbf{D}_{ij}^{\text{sub}}$ is the $h - BN$ substrate induced DMI exchange for layers 1 and 4 mediating the intra-layer exchange between spins i and j, with unit vector $\left(\hat{x} \times v_{ij}\right)$, where v_{ij} is the interaction unit vector between spins i and j. We approximate the two bilayer layer systems (layers 1-2 and layers 3-4) as being "pristine" monoclinic configuration CrI_3. The exchange interactions modulated by the Moiré geometry follow the slide-mapping method developed in (*7*), where the isotropic and DMI exchanges for layers 2 and 3 now contain a locational dependence, $\mathbf{r}$. This gives the full spin Hamiltonian for the atomistic system:

$$\begin{aligned}\mathcal{H} = -\frac{1}{2}\sum_{ij}\mathbf{S}_i J_{ij}^{lm}\left(\mathbf{r}\right)\mathbf{S}_j \; - \sum_{ij}\mathbf{D}_{ij}^{lm}\left(\mathbf{r}\right)\left(\mathbf{S}_i \times \mathbf{S}_j\right) \; - \sum_{ij}\mathbf{D}_{ij}^{\text{sub}}\left(\mathbf{S}_i \times \mathbf{S}_j\right) \\ -K_{2\perp}\sum_i \left(\mathbf{S}_i^z\right)^2 - \mu_B \mathbf{B}^{app} + \frac{\mu_0}{4\pi}\sum_{p\neq q}\frac{3\left(\mathbf{m}^q \cdot \hat{\mathbf{r}}\right)\hat{\mathbf{r}} - \mathbf{m}^q}{r^3}\end{aligned} \qquad (22)$$

$K_{2\perp}$ is the single-ion out-of-plane anisotropy, and $\mathbf{B}^{app}$ is the applied external field. The dipole field interaction is given by the macrocell approximation, with $\hat{\mathbf{r}}$ the vector distance between the moment m in cells p, q. Though our group in previous atomistic studies of CrI_3 and other 2D magnets has included Kitaev and biquadratic exchange interactions into the simulations (*8–11*), we do not make use of these exchanges in this study, due the lack of available data on the effect of the Moiré geometry on the atomistic parameters. Instead, the single-ion anisotropy is held constant and out of plane.

The Moiré lattice causes a modulation of the J_{ij}^{23} exchange constant between FM and AFM coupling and the $\mathbf{D}_{ij}^{lm}$ exchange constant between in-plane (IP) and out-of-plane (OOP) directions. This modulation, relative to the geometric Moiré lattice, is shown in Fig. S 40 for 0.5 and 1.1° twist angles. Specifically, Fig. S 40 shows the ratio of AFM to FM inter-layer exchange (bottom) and the ratio of in-plane to out-of-plane inter and intra layer DMI (top). Thus, pale regions on the figure correspond to areas with high spin frustration, which are concentrated

around the rhombohedral (AB) stacking configuration regions. The substrate DMI acts as a constant addition to the exchange matrix, but only for layers 1 and 4.

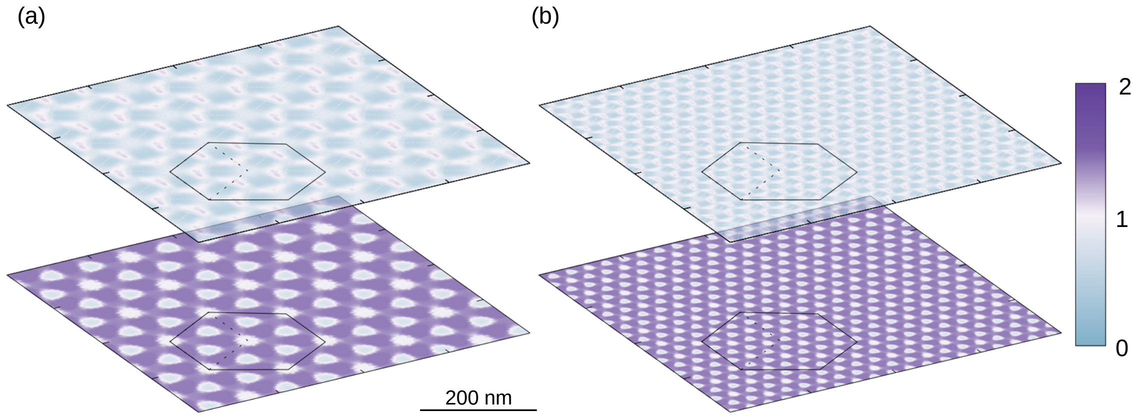

Figure S 40: (Bottom): ratio of AFM to FM interlayer exchange. (Top): ratio of IP to OOP inter and intralayer DMI. (a): 0.5° Moiré twist. (b): 1.1° Moiré twist. Dashed rhombus shows the Moiré unit cell for 0.5° twist. Solid hexagon shows the Moiré superlattice periodicity for the 0.5° twist.

In order to produce the correct FM (AFM) ordering of the AA and AB (AB') configurations, the constants produced from the Yang et al. method are rescaled by the pristine configuration calculations in Soenen et al. (*12*). Thus, the periodicity and shape of the modulation from the slide-mapping technique in Yang et al. (*7*) is able to be applied to any arbitrary Moiré rotation while matching the FM and AFM ordering seen in the AA, AB, and AB' regions.

To compare the simulation results better with the experimental imaging technique, we reduce the atomic scale resolution of the system by first boxing the atomistic magnetization into 5nm x 5nm microcell regions, then smoothing the results with gaussian interpolation having a characteristic decay of 30 nm. Due to the effect the Moiré lattice geometry has on the exchange constants, a single non-reproducible Moiré cell contains 800,00 to 3,000,000 spins (increasing with twist angle), with commensurate unique exchange constants. Thus, the computational

AB'	$1NN$	$2NN$	$3NN$	Unit
J_{ij}^{lm}	0.7585*	-0.5569*	-0.1925*	meV/link
J_{ij}^{ll}	3.9494	1.0456	-0.1216	
$\lvert D_{ij}^{ll}\rvert$	0.0806	0.0	0.1897	
$\lvert D_{ij}^{lm}\rvert$	0.0	0.0	0.0	

AB	$1NN$	$2NN$	$3NN$	Unit
J_{ij}^{lm}	0.435	0.655	0.515	meV/link
J_{ij}^{ll}	3.9207	1.0613	-0.1631 (-0.1088)	
$\lvert D_{ij}^{ll}\rvert$	0.0712 (0.0812)	0.0	0.1922 (0.1244)	
$\lvert D_{ij}^{lm}\rvert$	0.0	0.0873	0.0	

AA	$1NN$	$2NN$	$3NN$	Unit
J_{ij}^{lm}	0.1543	0.0184	-0.0767 (-0.2790)	meV/link
J_{ij}^{ll}	3.8893	1.0412	-0.0786	
$\lvert D_{ij}^{ll}\rvert$	0.0705	0.1187	0.07324	
$\lvert D_{ij}^{lm}\rvert$	0.0732	0.0	0.0135 (0.0361)	

Parameter	Value	Unit
μ_s	2.98	μ_B
$k_{2\perp}$	0.0135	meV/atom
D_{ij}^{sub}	0.195*	meV
T_N	$20-60$	K

Supplementary Table 2: Selected exchange constants and simulation parameters for the atomistic spin Hamiltonian for high-symmetry CrI_3 bilayer configurations. Parenthesis show values for alternate Cr lattice site due to broken exchange symmetry. The DMI vector for each interaction is determined by the calculated D_x, D_y, D_z value in (*7*). *Note: for the inter-layer exchange in the AB' configuration, 1NN, 2NN, and 3NN represent cutoff distances rather than neighbor locations.

cost and memory requirements limits larger simulation sizes to open boundary conditions only. Thus, textures at the edge of the lattice (< 20 nm) are ignored. In addition, the closeness of the simulated lattice size to the experimentally observed super-Moiré periodicity (400 nm vs. ∼200 nm) makes analysis of the simulated results using the auto-correlation function in the main text unavailable. Moreover, given the complexity of Eq.22 with several exchange interactions (1NN, 2NN, 3NN) along the interlayer and intralayer directions, as well as intrinsic and

interfacial DMI induced by the substrate (hBN) (*13*), anisotropy constants ($K_{2\perp}$), and dipolar interactions $\mathbf{B}^{app}$ (see Supplementary Table 2), which is calculated throughout the entire system self-consistently, an analytical solution of a such model is not practical. All these interactions are also modulated by the twist angle, which generates additional angular dependence, resulting in over 18,000,000 unique exchange tensors. The solution of this model can only be achieved numerically as showed here.

To ensure well-equilibrated MC results, we also perform several smaller scale field cooling simulations using the Landau-Lifshitz-Gilbert (LLG) equation activated using the Langevin thermostat to include thermal effects (*3*). We use a moderate damping value of $\alpha = 0.5$ to accelerate the simulations without compromising the dynamics. The magnetization using LLG techniques is shown in Fig. 46 with the evolution after cooling to 4 K for 1.5 ns. Since the spin system is especially exchange frustrated, we also compared our field cooling results and random-state MC results to MC simulations with a fully compensated, collinear AFM state. Fig. 41 shows an example simulation result for Moiré twist angle of 1.1 degree at 4 K after 600,000 MC steps. While the net magnetization for the two simulations is similar (row 3 from the top, labeled $\langle 4L \rangle$), only the random start simulation shows multiple domains and spin textures commensurate with experiment.

This quenched disorder, which in the context of the simulations corresponds to quench the system from a high-temperature state into a final one at a given temperature, resulted in a different - but yet similar - state. This happens if we start with a compensated state (fully AFM) with no domains, skyrmions, etc. This compensated state still moves to a highly non-collinear state, but the low temperature prevents an evolution to the domains/skyrmions we see in the experiments. In this aspect, by starting the calculations from a fully AFM configuration may drive the system to a meta-stable state which is far from the ground-state.

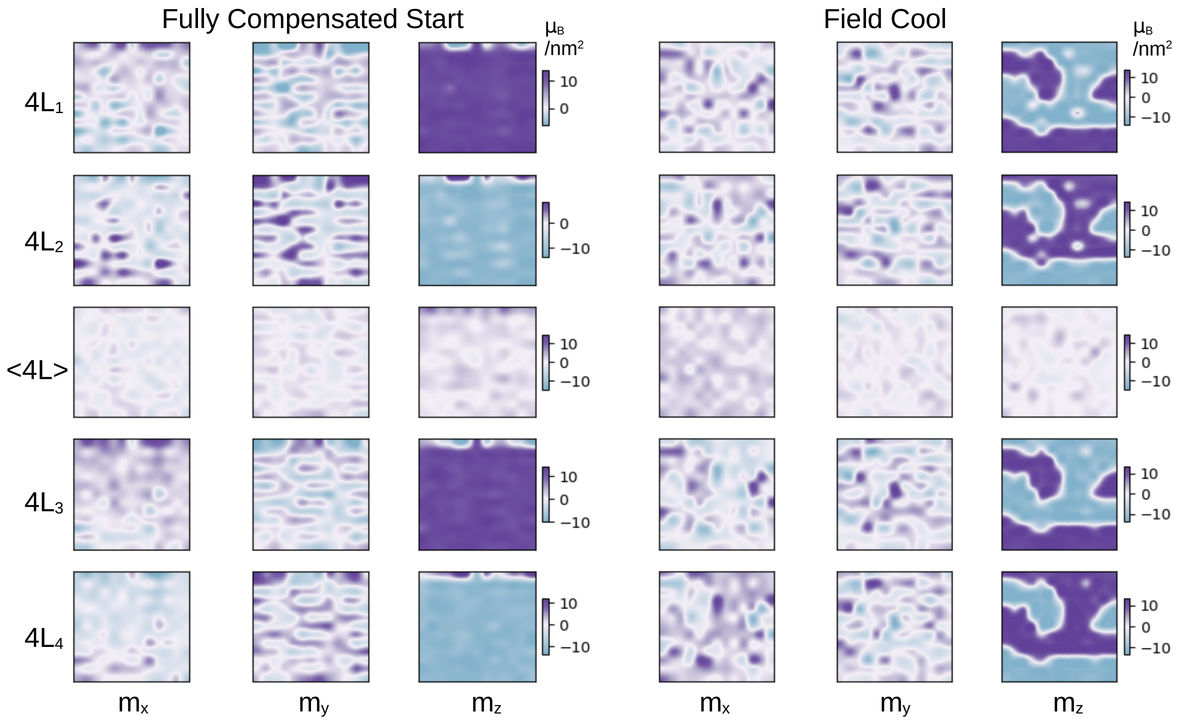

Figure S 41: 1.1° twist angle for MC simulations with random starting spins to mimic LLG field cooling (right) and collinear AFM spins (left).

19.2 Results

19.2.1 Moiré Spin Textures

The net magnetization in Fig. S 43 shows that the exchange frustration at the interface alone is enough to produce uncompensated moments across the AFM stack. However, due to the limited system size it is difficult to compute the stray fields commensurate with the experimental measurement. The net magnetization shown in the central row of the associated figures is thus only an approximation of the representative field measured by the experimental technique. Fig. S 42 compares a representative example of simulated texture for 0.5, 1.1, and 2.0° twist angles (see Fig. 1e-g) of the main text). Both the sublayer and net magnetization domain volume increase going from 0.5 to 1.1° twists, with only remnant saturation existing for the 2.0° twist

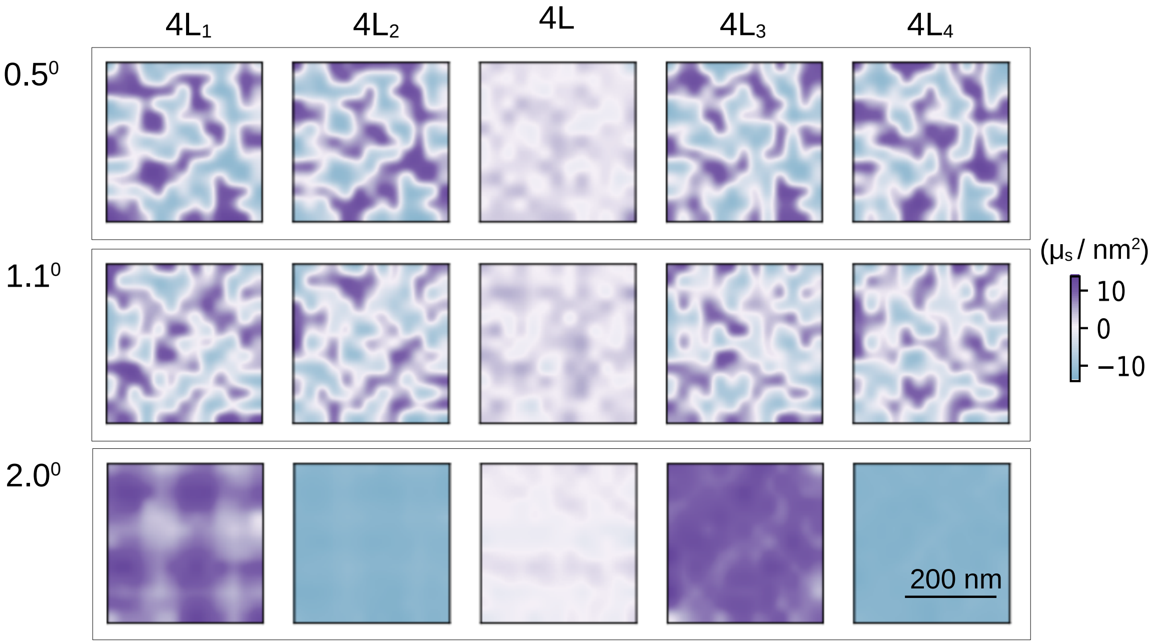

Figure S 42: 0.5°, 1.1°, and 2.0° Moiré twist angle simulation results showing the z component of the magnetization. The column labels $4L_i$ correspond to the magnetisation profile of each individual layer (with 4L the net profile).

giving a primarily AFM state.

The characteristic Moiré modulation of the magnetisation in the tB-CrI_3 follows the geometric Moiré periodicity, in strong contrast to the diffuse textures in Fig. 42. The triangular domains formed in the AFM regions of Fig. 44 are consistent with the earlier atomistic modeling of tB-CrI_3 in (*14*), though the scale of Fig. 44 does not resolve the highly localized topology present.

As discussed further along in Sec. 19.2.3, the sizable exchange frustration present between the 'pristine' bilayer CrI_3 and the Moiré interface is enough to nucleate spin textures and Bloch-type skyrmions even without DMI intrinsic to the interface and bilayer CrI_3, or DMI induced from the CrI_3/h-BN interface 43. A sizable difference is seen between the magnetisation texture without DMI in Fig. 43 and the texture with DMI in Fig. 42. The presence of DMI from the

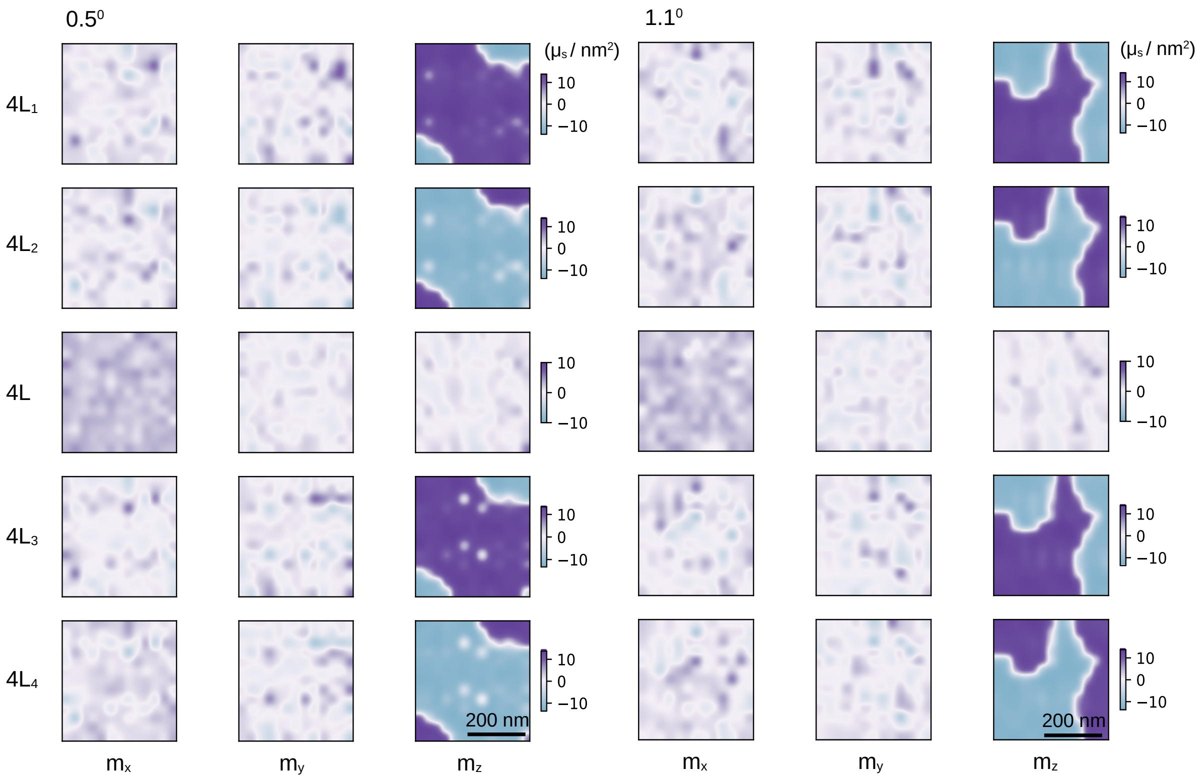

Figure S 43: 0.5° and 1.1° Moiré twist angle simulation results. The intrinsic and substrate DMI for all four layers has been set to zero. The row labels 4L$_i$ correspond to the magnetisation profile of each individual layer (with 4L the net profile). Each column shows the x, y, z component of the magnetisation in μ_B/nm^2.

CrI_3/h-BN interface does not seem to affect the size of the domains, but rather stabilizes the Bloch-type skyrmions from the exchange frustration into Néel type skyrmions. The size of the domains varying with DMI magnitude, direction, etc. has not yet been quantified due to the size of the variable active space and computational costs.

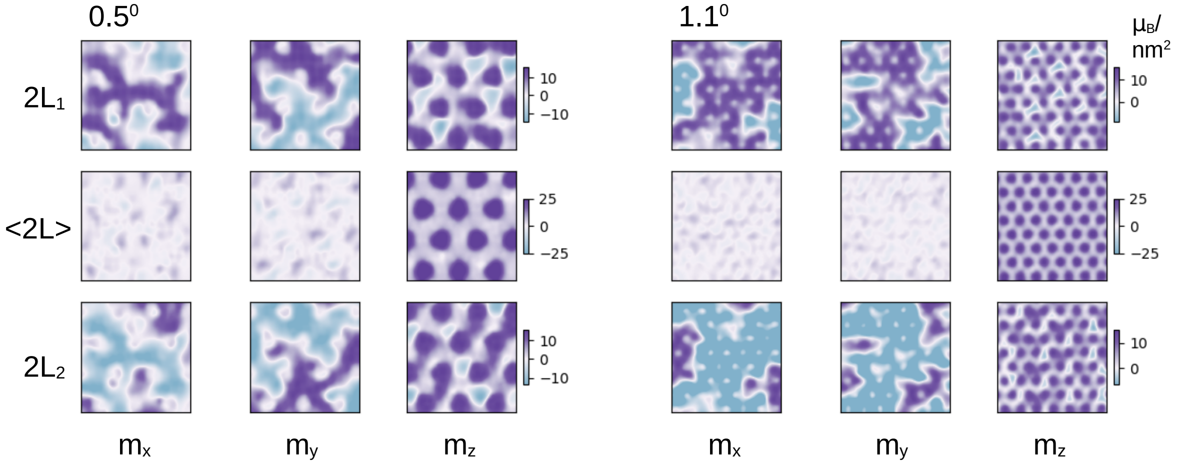

Figure S 44: (left): 0.5° twisted bilayer CrI_3. (right): 1.1° twisted bilayer CrI_3. Both simulations are 150nm x 150nm square with open boundary conditions, field cooled rapidly to 0 K.

19.2.2 Moiré Phases

For twist angles between 0.0° and 1.1°, we analyse the spin-collinearity leading to the remnant magnetisation across the four CrI_3 layers. For these simulations, we maintain the 510 mT field during equilibration to bias the domains away from the $-z$ axis which highlights the FM/AFM regions. To visualise the spin collinearity, we introduce a normalised cross correlation value with zero lag time of the magnetisation direction for the four layers. As with the autocorrelation function in Eq. 1 of the main text, we scale this value by $1/dr^3$ to account for decay in the spin correlation. This cross correlation value (CC) is calculated according to:

$$CC = \sum_{i,j} \sum_{l} \mathbf{M}_l(x_i, y_i) \cdot \mathbf{M}_l(x_j + y_j) \frac{1}{\left(\Delta x_{ij}^2 + \Delta y_{ij}^2\right)^{3/2}} \tag{23}$$

Thus, bright areas in the second row of Fig. S 45 represent highly-collinear regions across all four layers. In order to match these regions of collinearity with the remnant $\langle m_z \rangle$ magnetisation in row one, we scale the $\langle m_z \rangle$ value by the CC value in row two. To match the remnant magnetisation caused by non-collinear spins, row four shows the magnetisation in row

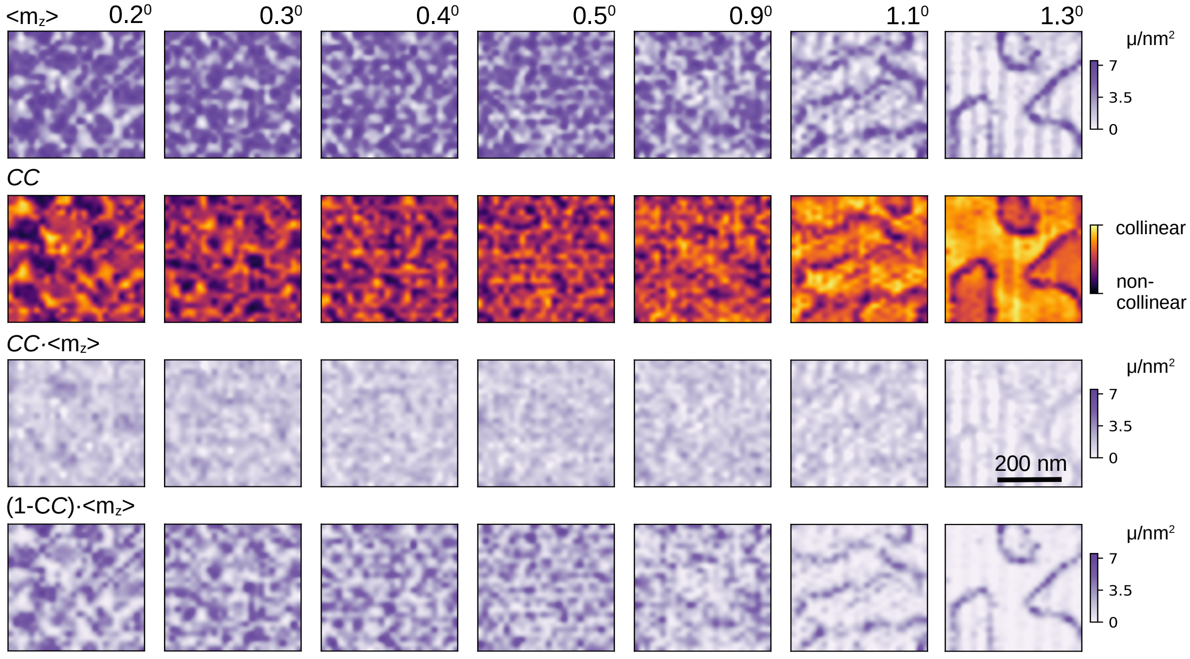

Figure S 45: (Row 1): Remnant magnetisation and corresponding spin CC (row 2) for increasing Moiré twist angles. (Row 3): remnant magnetisation scaled by the CC value in row 2. (Row 4): remnant magnetisation scaled by 1-CC value in row 2. Since the CC in row 2 is normalised to zero and one, a direct comparison between the μ/nm^2 value in row 3/4 and row 1 is possible. Dipolar interactions were not included in these simulations, which negligible variations are observed relative to their inclusion.

one scaled by one minus the CC value in row two.

For low twist angles ($\langle 0.4°$), this produces regions of remnant magnetisation matching with both collinear and non-collinear spins. Above this angle, the remnant collinear magnetisation diminishes into stochastic noise, while the remnant non-collinear magnetisation maintains clear domains which shrink with increasing twist angle. By the 0.9°/1.1° twist angles, the remnant magnetisation from non-collinear spins have fully merged into larger structures above the size of the AB' regions in the Moiré supercell. This suggests the presence of three critical twist angles for the Moiré supercell magnetisation: α_1, where the collinear domains disappear from the remnant magnetisation ($\approx 0.4°$ in our simulations); α_2, where the non-collinear domains

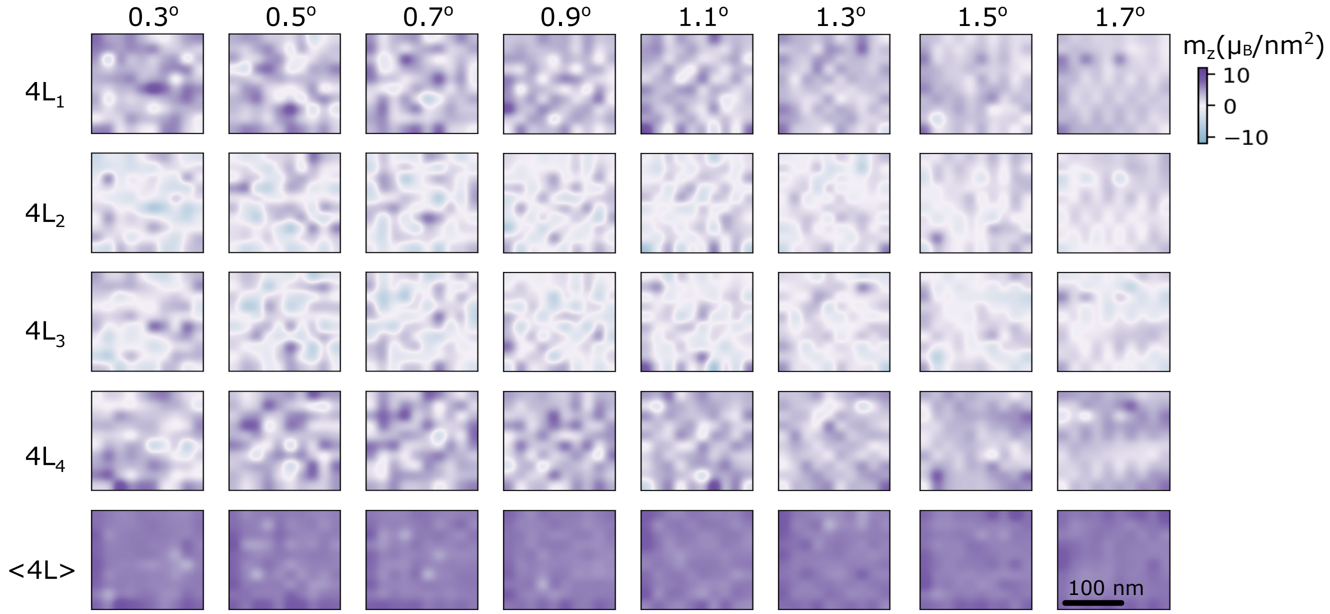

Figure S 46: Layer resolved m_z for increasing twist angle. Data simulated using the LLG equation including dipolar interactions as in Eq.22. The spins evolved under 510 mT field rapid cooling to 4 K. The row labels 4L$_i$ correspond to the magnetisation profile of each individual layer (with 4L the net profile). Each column shows the z component of the magnetisation in μ_B/nm^2 for increasing twist angle.

following the Moiré superlattice merge to form larger textures ($\approx 0.9°$ in our simulations); α_3, where the surviving remnant textures disappear into random domains ($> 1.3°$ in our simulations). Since these critical angles depend on the ratio of FM to AFM domain energy for each Moiré twist angle, they are naturally dependent on the exchange and anisotropy constants used in the simulations. Thus, a different set of constants in Table 2 may adjust these angles. Simulations using LLG equation with Langevin thermostat ((*3*)) were used to support the MC results 46.

We analyze the component energies of the field cooled systems using 50,000 MC steps. The total exchange and anisotropy energies in Fig. 49 likewise suggest three Moiré phase regions: (I) domains following the geometric Moiré lattice, (II) super Moiré domains with length scale

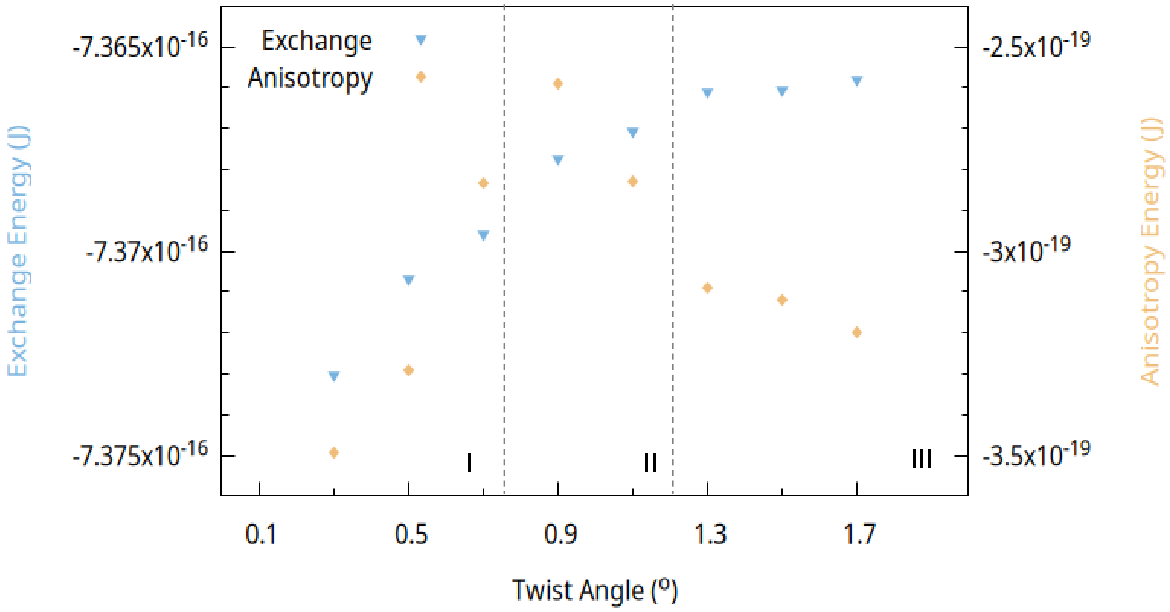

Figure S 47: (blue): Sum of the isotropic and anisotropic exchange components in the atomistic Hamiltonian. (gold): sum of the single ion anisotropy component in the atomistic Hamiltonian. The regions (I), (II), and (III) suggest different phases in the Moiré domains.

larger than the geometric Moiré wavelength, and (III) fully AFM domains with no long-range ordering. The anisotropy has its maximum (least stable) value for twist angles near 0.9°, showing the greater tendency for in-plane orientation, characteristic of the large exchange frustration and DMI of the tDB-CrI_3 (Fig. 40). To compare the effects of the frustration present in the tDB-CrI_3 relative to the Moiré twist interface, we also perform MC simulations on twisted-bilayer (tB) CrI_3 (Fig. 44). Here, the tB directly follows the geometric Moiré modulation with no super-Moiré texture present. Likewise, the exchange at the interface layers is largely compensated and AFM along the x, y directions (note the significantly smaller M_x and M_y μ_B/nm^2 values compared to the nearly fully saturated magnetisation along M_z for both twist angles).

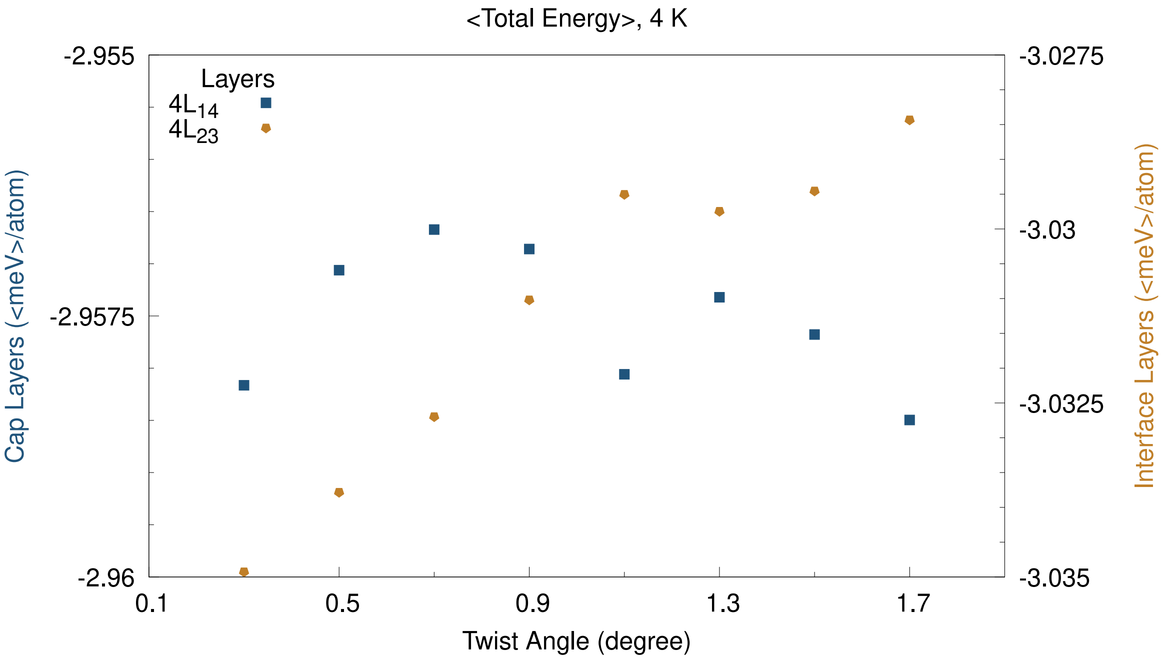

Figure S 48: (blue): total energy of the cap layers of CrI_3 (layers 1 and 4). (gold): (blue): total energy of the interface layers of CrI_3 (layers 2 and 3). Simulation at 4 K averaged over 50,000 MC steps.

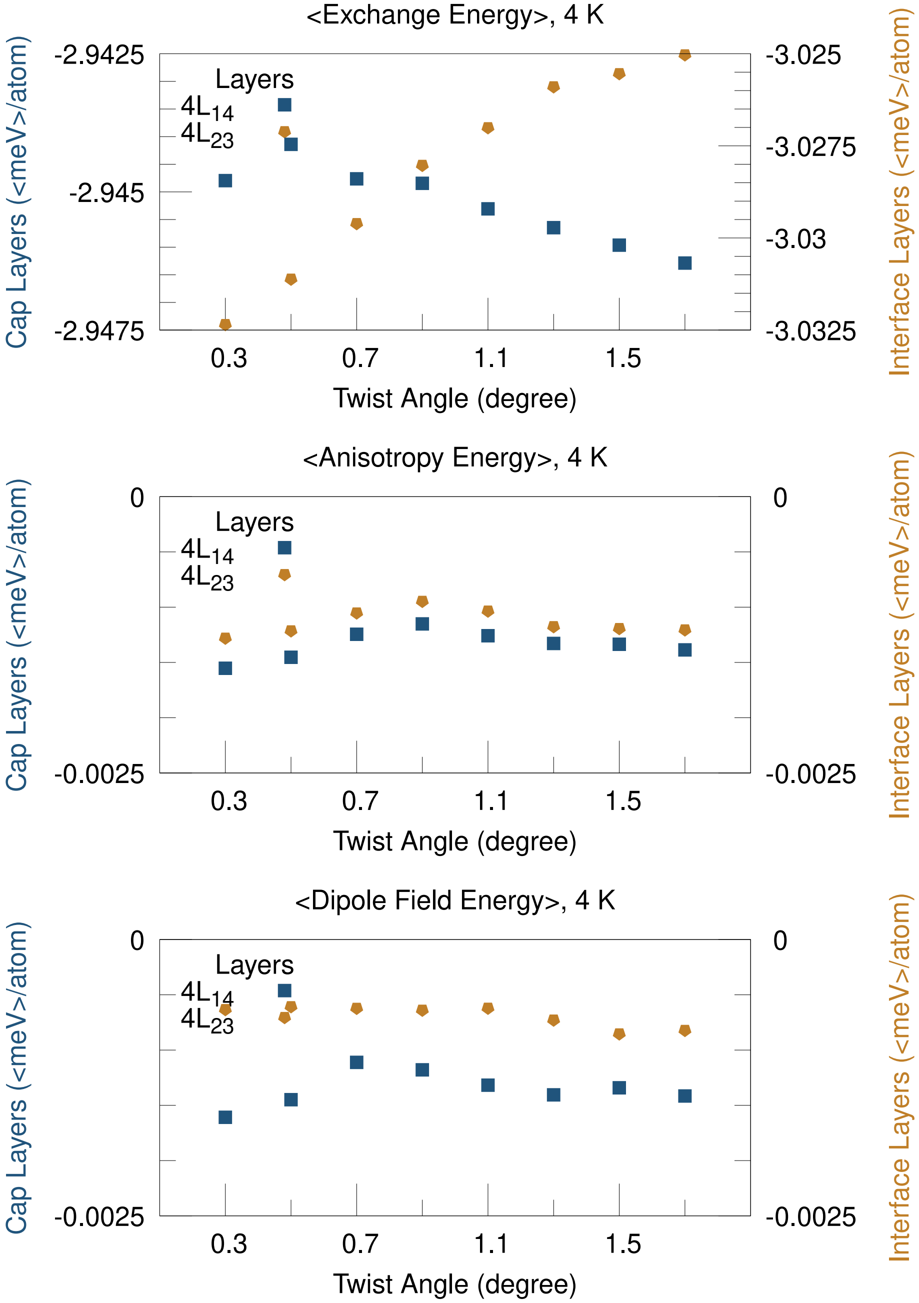

Figure S 49: Energy components of the cap layers of CrI_3 (layers 1 and 4) in blue. (gold): (blue): component energies of the interface layers of CrI_3 (layers 2 and 3). Simulation at 4 K averaged over 50,000 MC steps.

19.2.3 Skyrmion Formation

The sizable exchange frustration generated by the AB/AA to AB' configuration transitions in the Moiré lattice (Fig. 40) is enough to nucleate non-collinear domains, even without DMI affecting the exchange. These stabilize into Bloch-type skyrmions/antiskyrmions at the boundaries of the AB' (AFM) locations (Fig. S 50). At larger twist angles, the AFM region volume shrinks, diminishing the size of the textures below the 30 nm resolution (Fig. S 43).

The non-collinear textures in Fig. S 50 show Bloch-type, Neél-type, and pair skyrmions arising from the exchange frustration at the Moiré interface. The textures exist purely in layers 3/4 (1/2) in order to stabilize the interface.

The inclusion of DMI into the system greatly stabilizes the presence of additional spin textures. This can take the form of substrate induced DMI (acting only on layers 1 and 4) or the intrinsic DMI (acting on all layers), though only the intrinsic DMI between interface layers 2 and 3 is modulated by the Moiré lattice. Increasing the substrate DMI from 0.195 meV to 0.585 meV still supports texture generation, but does not shift the character away from Bloch-type and Neél-anti-type skyrmions.

We note that the skyrmions present for both 0.5 and 1.1° twist angles (Figs. S 51, S 52) occupy different volumes of the AFM coupled layers due to asymmetric exchange present from the substrate DMI in layers 1(4) and the exchange frustration and DMI modulation present at the Moiré interface. This naturally gives an uncompensated moment which would present as a stray field.

The addition of intrinsic DMI to the system significantly weakens the domain energy of the intralayer FM coupling, allowing for more spin texture to occur during the field cooling. The average topology of these domains calculates to zero, but some topologically protected spin textures remain from the exchange frustration at the twist interface. Generally we find these textures exhibit Bloch-like topology, commensurate with the parity between IP and OOP DMI

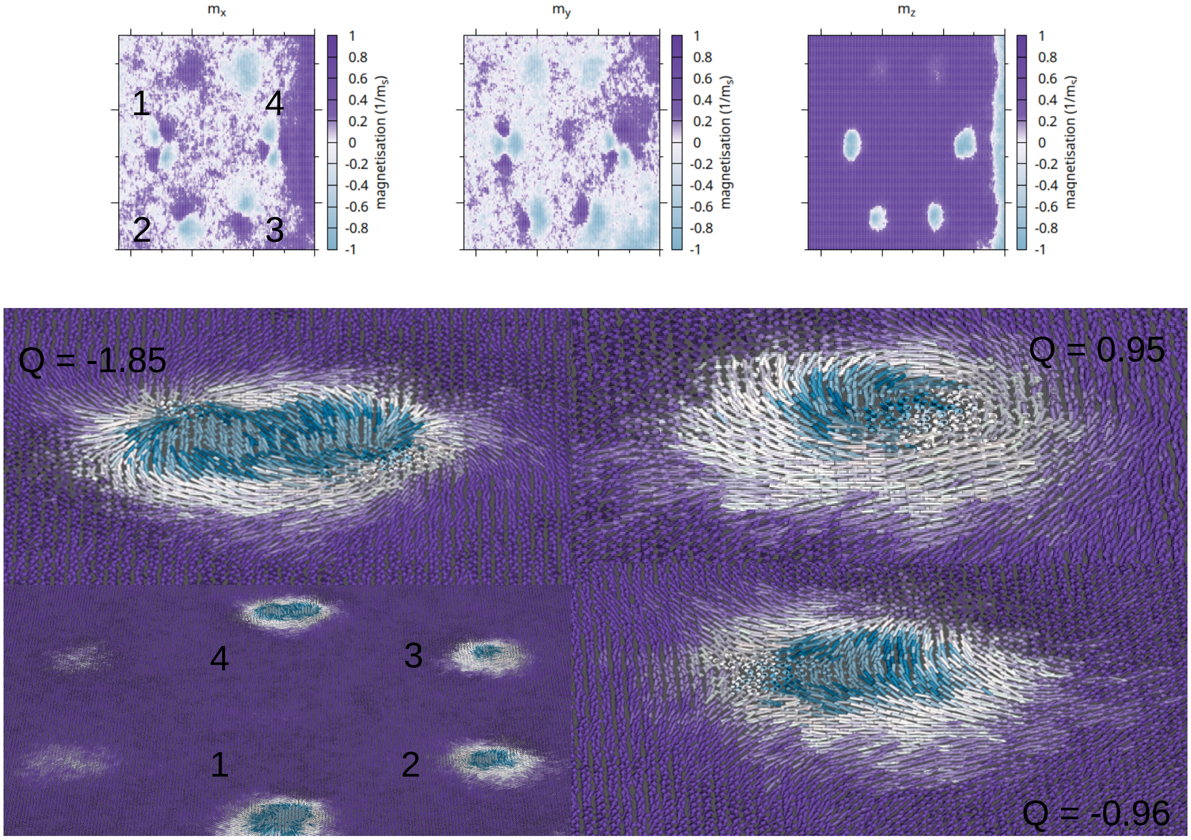

Figure S 50: (Top): sublattice magnetization for a collection of spin textures. (Bottom): visualization of the corresponding spin textures with their calculated topological charge.

at the exchange frustrated regions (see Fig. S 40). Inclusion of the substrate DMI, however, sees stabilization of Neél-type skyrmions (Fig. S 51). For 0.5° twist, the required substrate DMI to stabilize the Neél-type texture is 0.585 meV. The 1.1° twist–with its smaller skyrmion volume–requires less substrate DMI at 0.195 meV (Fig. S 52). Both simulation results show the trend of increasing domain size with increasing twist angle.

Also present in the simulation results for 1.1° twist angles are combined skyrmion bubble textures formed over multiple AFM configuration regions, shown for example in Fig. S 53. There, the topological charge value is 1.21, showing incomplete absorption of a set of skyrmion-

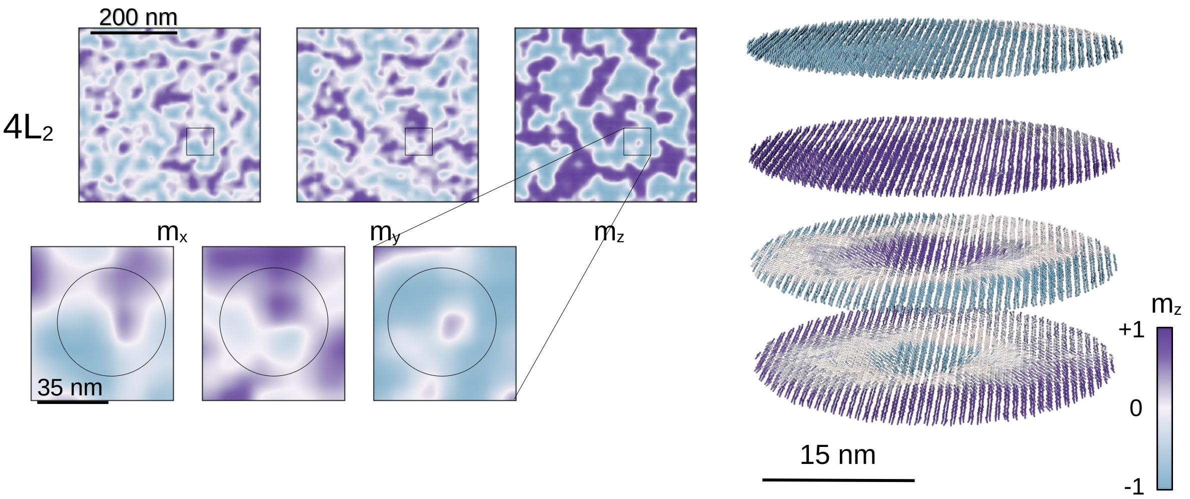

Figure S 51: 0.5° twist localized skyrmion with topological charge of 0.86. The intrinsic DMI has values in table 2, with the substrate DMI having a total contribution of 0.585 meV. The unequal DMI and exchange frustration between layers 1 and 2 leads to differently sized topology giving the skyrmion an uncompensated moment.

antiskyrmion pairs. Unlike the skyrmions above nucleated by high frustration configuration sites and stabilized by the substrate DMI, this texture exists on all four levels, though some asymmetry between layers 1/2 and 3/4 still exist, and it stabilized by the domain energy from the FM intralayer exchange.

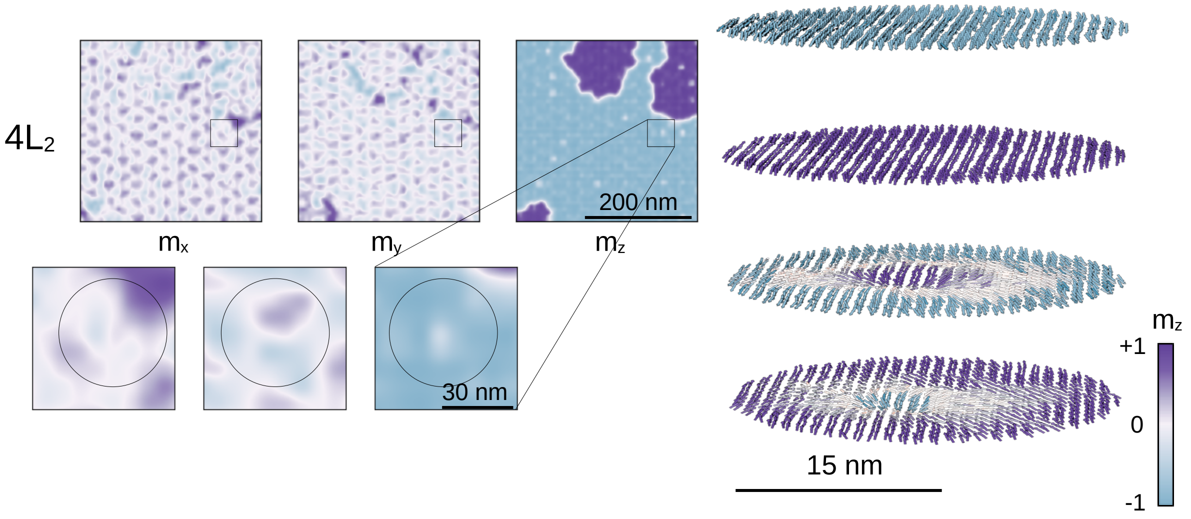

Figure S 52: 1.1° twist localized skyrmion with topological charge of 0.89. The intrinsic DMI has values in table 2, with the substrate DMI having a total contribution of 0.195 meV. The unequal DMI and exchange frustration between layers 1 and 2 leads to differently sized topology giving the skyrmion an uncompensated moment.

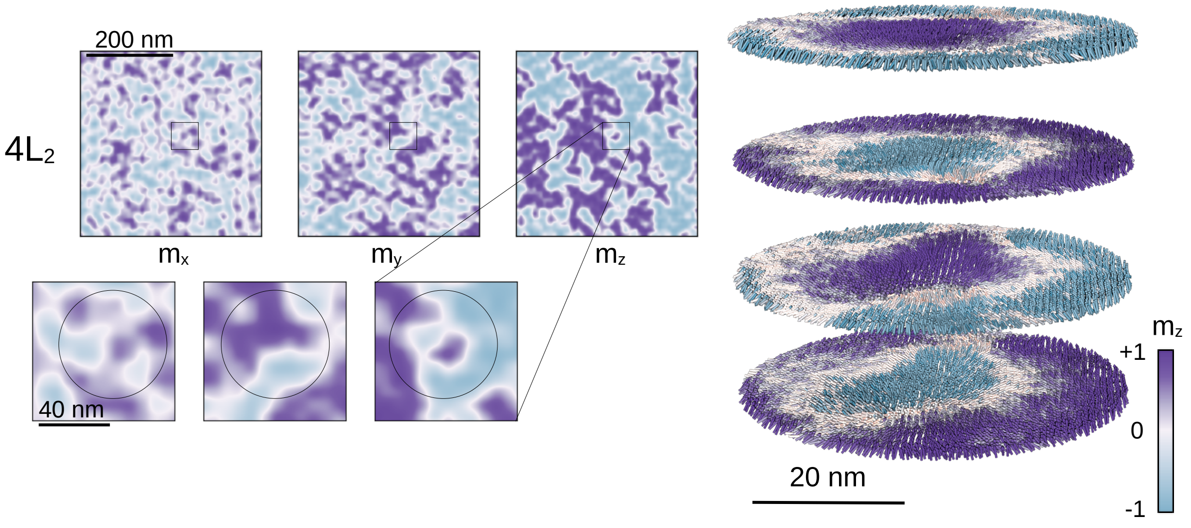

Figure S 53: 1.1° twist localized skyrmion with topological charge of 1.21. The size of the texture encompasses multiple high exchange frustration regions leading to a merging of the texture and presence of the skyrmion across all four layers.

References

1. M. W. Doherty, *et al.*, The nitrogen-vacancy colour centre in diamond, *Physics Reports* **528**, 1 (2013).

2. S. Tan, Y. P. Ma, I. M. Thomas, J. Wikswo, Reconstruction of two-dimensional magnetization and susceptibility distributions from the magnetic field of soft magnetic materials, *IEEE transactions on magnetics* **32**, 230 (1996).

3. R. F. L. Evans, *et al.*, Atomistic spin model simulations of magnetic nanomaterials, *Journal of Physics: Condensed Matter* **26**, 103202 (2014).

4. J. D. Alzate-Cardona, D. Sabogal-Suárez, R. F. L. Evans, E. Restrepo-Parra, Optimal phase space sampling for Monte Carlo simulations of Heisenberg spin systems, *Journal of Physics: Condensed Matter* **31**, 095802 (2019).

5. J. Meseguer-Sánchez, *et al.*, Coexistence of structural and magnetic phases in van der Waals magnet CrI3, *Nature Communications* **12**, 6265 (2021).

6. F. Cantos-Prieto, *et al.*, Layer-dependent mechanical properties and enhanced plasticity in the van der Waals chromium trihalide magnets, *Nano Letters* **21**, 3379 (2021).

7. B. Yang, Y. Li, H. Xiang, H. Lin, B. Huang, Moiré magnetic exchange interactions in twisted magnets, *Nature Computational Science* **3**, 314–320 (2023).

8. D. A. Wahab, *et al.*, Quantum Rescaling, Domain Metastability, and Hybrid Domain-Walls in 2D CrI3 Magnets, *Advanced Materials* **33** (2020).

9. M. Augustin, S. Jenkins, R. F. L. Evans, K. S. Novoselov, E. J. G. Santos, Properties and dynamics of meron topological spin textures in the two-dimensional magnet CrCl3, *Nature Communications* **12** (2021).

10. A. Kartsev, M. Augustin, R. F. L. Evans, K. S. Novoselov, E. J. G. Santos, Biquadratic exchange interactions in two-dimensional magnets, *npj Computational Materials* **6**, 150 (2020).

11. J. Macy, *et al.*, Magnetic field-induced non-trivial electronic topology in Fe3xGeTe2, *Applied Physics Reviews* **8**, 041401 (2021).

12. M. Soenen, C. Bacaksiz, R. M. Menezes, M. V. Milošević, Stacking-dependent topological magnons in bilayer, *Physical Review Materials* **7** (2023).

13. F. Zhang, *et al.*, Strong Dzyaloshinskii-Moriya interaction in monolayer $\mathrm{CrI_3}$ on metal substrates, *Phys. Rev. B* **106**, L100407 (2022).

14. D. Ghader, B. Jabakhanji, A. Stroppa, Whirling interlayer fields as a source of stable topological order in moiré CrI3, *Communications Physics* **5** (2022).
